# Comparative transcriptome and metabolome analyses provide new insights into the molecular mechanisms underlying taproot thickening in *Panax notoginseng*

**DOI:** 10.1186/s12870-019-2067-5

**Published:** 2019-10-26

**Authors:** Xue-Jiao Li, Jian-Li Yang, Bing Hao, Ying-Chun Lu, Zhi-Long Qian, Ying Li, Shuang Ye, Jun-Rong Tang, Mo Chen, Guang-Qiang Long, Yan Zhao, Guang-Hui Zhang, Jun-Wen Chen, Wei Fan, Sheng-Chao Yang

**Affiliations:** 1 State Key Laboratory of Conservation and Utilization of Bio-resources in Yunnan, The Key Laboratory of Medicinal Plant Biology of Yunnan Province, National& Local Joint Engineering Research Center on Germplasm Innovation & Utilization of Chinese Medicinal Materials in Southwest China, Yunnan Agricultural University, Kunming, 650201 China; 2 College of Horticulture and Landscape, Yunnan Agricultural University, Kunming, 650201 China; 3 State Key Laboratory of Plant Physiology and Biochemistry, College of Life Sciences, Zhejiang University, Hangzhou, China

**Keywords:** *Panax notoginseng*, Taproot thickening, Signal transduction, Metabolic regulation

## Abstract

**Background:**

Taproot thickening is a complex biological process that is dependent on the coordinated expression of genes controlled by both environmental and developmental factors. *Panax notoginseng* is an important Chinese medicinal herb that is characterized by an enlarged taproot as the main organ of saponin accumulation. However, the molecular mechanisms of taproot enlargement are poorly understood.

**Results:**

A total of 29,957 differentially expressed genes (DEGs) were identified during the thickening process in the taproots of *P. notoginseng.* Gene Ontology and Kyoto Encyclopedia of Genes and Genomes pathway enrichment revealed that DEGs associated with “plant hormone signal transduction,” “starch and sucrose metabolism,” and “phenylpropanoid biosynthesis” were predominantly enriched. Further analysis identified some critical genes (e.g., RNase-like major storage protein, DA1-related protein, and Starch branching enzyme I) and metabolites (e.g., sucrose, glucose, fructose, malate, and arginine) that potentially control taproot thickening. Several aspects including hormone crosstalk, transcriptional regulation, homeostatic regulation between sugar and starch, and cell wall metabolism, were identified as important for the thickening process in the taproot of *P. notoginseng*.

**Conclusion:**

The results provide a molecular regulatory network of taproot thickening in *P. notoginseng* and facilitate the further characterization of the genes responsible for taproot formation in root medicinal plants or crops.

## Background

Root/stem thickening presents as an enlarged structure in some plant species where it is used as a storage organ for nutrients, thus providing food for humans and animals. Root/stem thickening is mainly regulated by the vascular system as a consequence of the interaction between the primary and secondary cambium. The primary cambium determines the primordium formation of the root/stem, while the secondary cambium affects root/stem thickening by increasing cell number and volume [1]. Studies have shown that signal regulation and metabolic change are the main endogenous factors during root/stem thickening, while environmental factors such as soil temperature, humidity, light, photoperiod, carbon dioxide, and drought also affect this process [2–6]. A comprehensive understanding of the molecular mechanisms regulating various aspects of root/stem thickening is required to facilitate the development of new plant varieties.

“Omics” technologies have contributed to our understanding of the molecular mechanisms underlying root/stem thickening. The fleshy taproot of radish (*Raphanus sativus*) is an important storage organ that determines radish yield and quality. Genome sequencing and transcriptome analysis indicated that the genes related to carbohydrate metabolism were prominently activated during taproot thickening [7, 8]. Proteomic analysis identified that cell division cycle 5-like protein (CDC5), expansin B1 (EXPB1), and xyloglucan endotransglucosylase/hydrolase protein 24 (XTH24) were responsible for the cell division and expansion of taproot thickening in radish [9]. Further high-throughput sequencing of small RNA indicated that microRNAs were also specific at different developmental stages, and their targets encoding transcription factors (TFs) and other functional proteins, including nuclear TF Y subunit A-2 protein (NF-YA2), IAA-amino acid hydrolase ILR1 gene (ILR1), basic helixloop-helix TF bHLH74 (bHLH74), CLAVATA3/ESR-related 41 (CLE41), XTH16 and EXPA9 were involved in radish taproot thickening [10]. In onion (*Allium cepa*), the rapid swelling of the bulb results in a significantly decreased sucrose content at an early stage of development, whereas fructose and glucose contents increase significantly at the mature stage, suggesting that sucrose metabolism plays an important role in onion bulb formation [11]. Additionally, Wang [12] reported that root tuberization in cassava (*Manihot esculenta*) was correlated with the significant up-regulated expression of carbohydrate metabolic enzymes. Of these, the 14–3-3 proteins and their binding enzymes might play important roles in carbohydrate metabolism and starch accumulation during cassava root tuberization. Similar results were also reported in potato (*Solanum tuberosum*) [13]. These results indicated that root/stem thickening is a complex trait that is controlled by multiple biological processes/pathways. However, these studies mainly focused on crops, and there is still a lack of understanding of the root/stem thickening process in other plants, particularly medicinal plants.

The enlarged root, stem and rhizome in medicinal plants are important medicinal organs that accumulate numerous active ingredients and account for one-third of the species recorded in the Chinese Pharmacopoeia. At present, many studies have aimed to elucidate the relationship between the development of enlarged tissues and the accumulation of secondary metabolites. For example, comparative transcriptome studies between tuberous roots and shoots in *Aconitum heterophyllum* showed that 3-hydroxy-3-methylglutaryl-CoAreductase (HMGR), mevalonate kinase (MVK), mevalonate diphosphate decarboxylase (MVDD), and 1-hydroxy-2-methyl-2-(E)-butenyl 4-diphosphate synthase (HDS) were required for aconite alkaloid biosynthesis, while genes encoding TFs, including bHLH, MYB basic leucine zipper (bZIP), and ATP-binding cassette (ABC) transporter, were implicated in tuberous root development [14]. In *Ophiopogon japonicus*, genes related to flavonoid and saponin synthesis were negatively regulated with tuberous root development, while genes related to polysaccharide synthesis were positively regulated in the early stage and negatively regulated in the later stage [15]. However, these studies lack a systematic investigation of the enlargement of these tissues.

*Panax notoginseng* (Burk) F.H.Chen is one of the most famous Chinese traditional medicinal plants belonging to the *Panax* genus in the Araliaceae family [16]. Triterpenoid saponins are the main active components that are widely used in the treatment of stroke injury, coronary heart disease, angina pectoris, cerebrovascular sequelae, hypertension and other diseases in *P. notoginseng* [17–19]. Currently, *P. notoginseng* is the main raw material of more than 400 Chinese patented medicines with an annual demand of 8 million kilograms and a total output value of more than 70 billion RMB [20, 21]. Taproot thickening in *P. notoginseng* is responsible for saponin accumulation, thus directly affecting its quality and yield. Traditionally, the quality of *P. notoginseng* has mainly been evaluated according to the appearance and size of the taproot, while the harvest period is primarily determined based on the taproot yield. However, there is a lack of understanding of the molecular mechanisms regulating taproot morphogenesis in *P. notoginseng*. In this study, the expression profiles of key stages during taproot thickening were constructed using one-year-old *P. notoginseng* plants. Further integrated transcriptomic and metabolomic analysis revealed the regulatory networks and critical genes potentially controlling taproot thickening. These results should serve as an important public reference platform for identifying the functions of candidate genes, thus facilitating the dissection of the molecular mechanisms underlying taproot development in *P. notoginseng*.

## Results

### Morphological changes during *P. notoginseng* taproot thickening

**Fig. 1:**
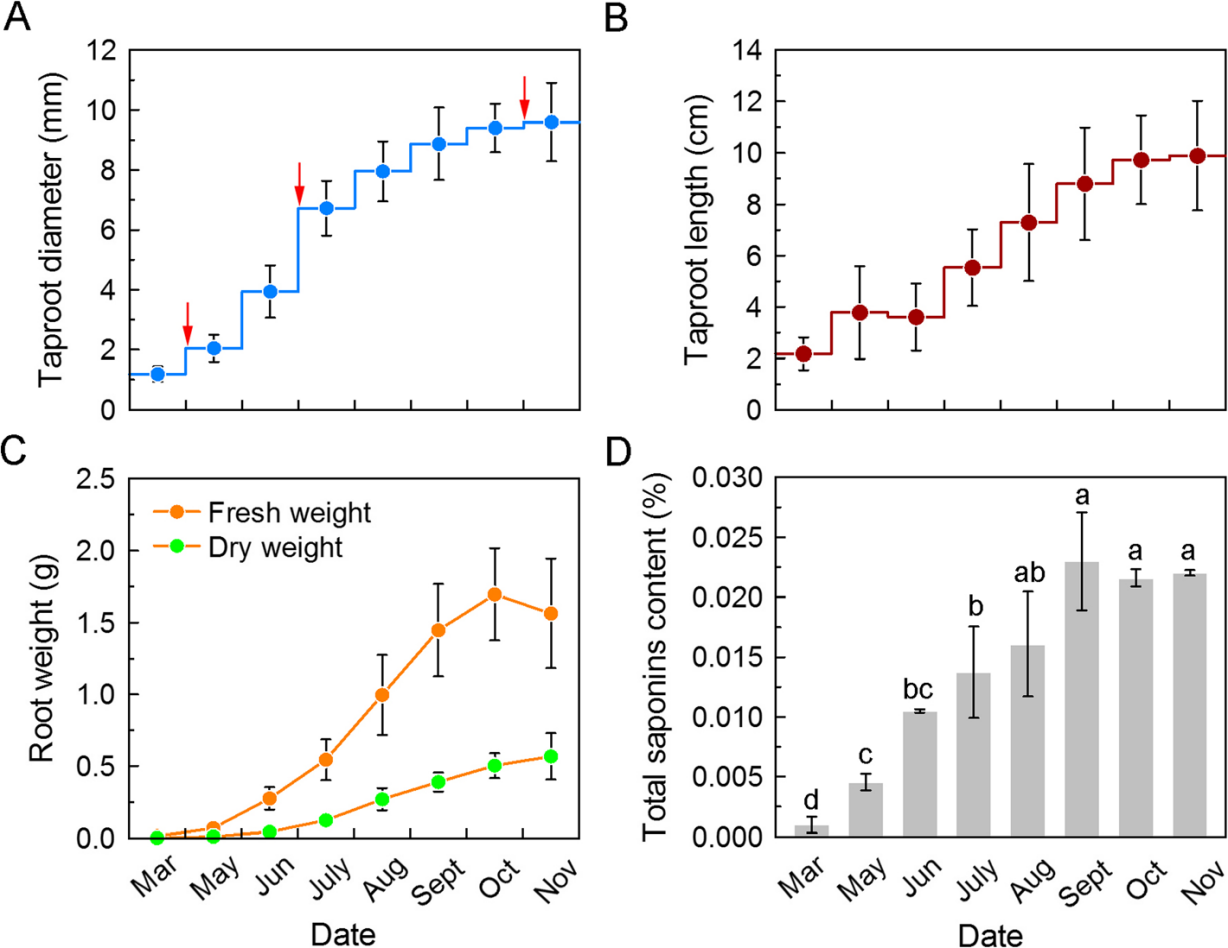
Determination of growth indices of *P. notoginseng* taproots at different developmental stages. **a** Taproot diameter. **b** Taproot length. **c** Taproot weight. **d** Total saponins content. Data are expressed as means ± SD (*n* = 30). Columns with different letters are significantly different at *P* < 0.05

We measured the dynamic growth indexes of the taproots of one-year-old *P. notoginseng* plants monthly. The results showed that the seed root germinated in March, while thickening took place from May to July and stabilized from August to November (Fig. 1a). However, the taproot elongation was not significant until July in comparison with March (Fig. 1b). Both the fresh and dry weight of the taproot increased with the growth period, but there was no significant difference from August to November (Fig. 1c). Similarly, the total saponins also increased with the growth month, but there was no significant difference from September to November (Fig. 1d). This suggested that fresh weight is indicative of saponin content during the first year of *P. notoginseng* growth.

**Fig. 2:**
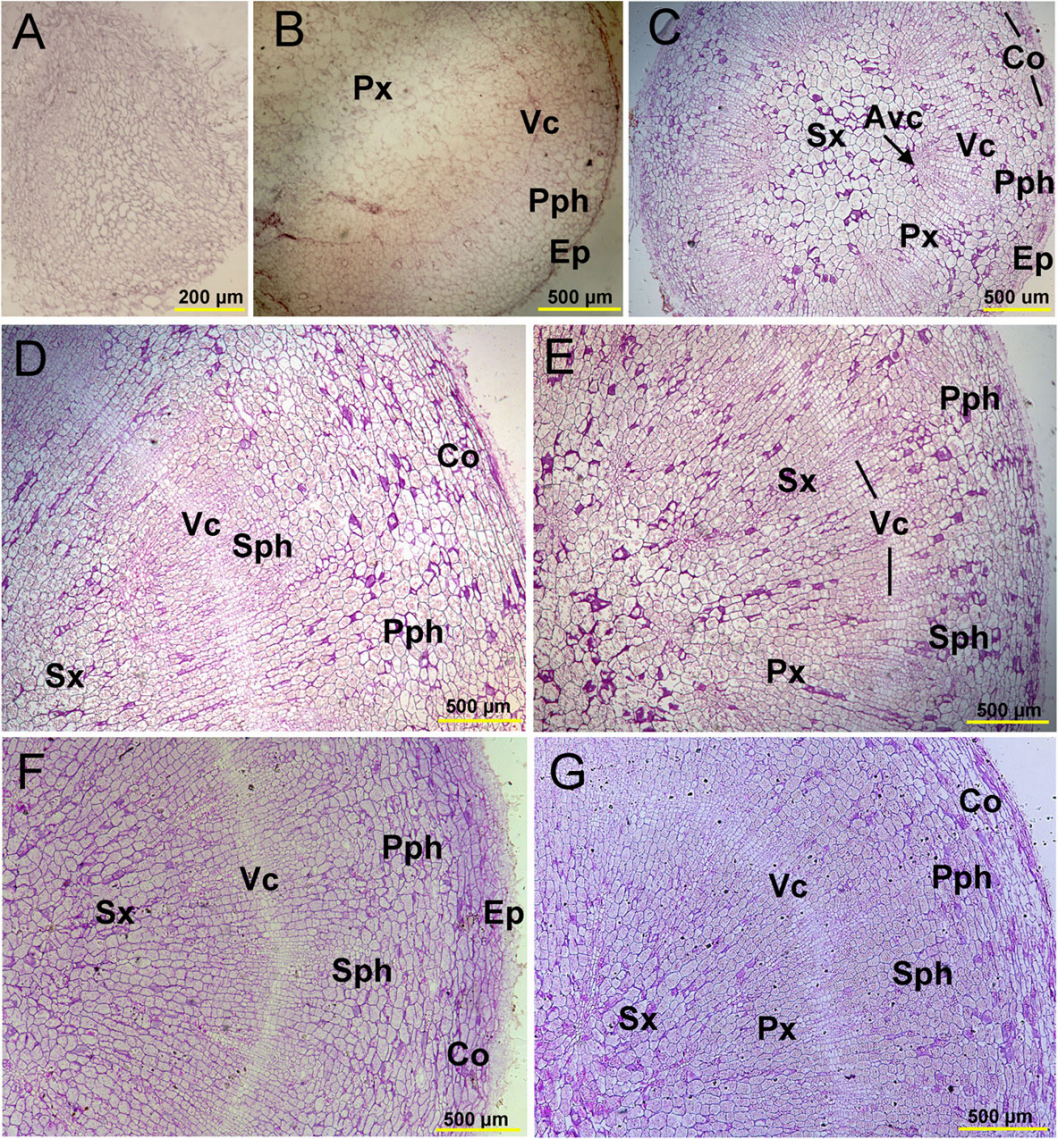
Anatomical structure of *P. notoginseng* taproots at different developmental stages. **a** March. **b-e** May to August. **f**, **g** October–November. Avc, accessory cambium. Co, cortex. Ep, epidermis. Ppc, phloem parenchyma cell. Pph, primary phloem. Px, primary xylem. Sph, secondary phloem. Sx, secondary xylem. Vc, vascular cambium

Taproot thickening is associated with secondary meristem activity. We observed the anatomical structure of the taproot during different months of growth. The results showed that the vascular bundles were not well developed in the seed root stage in March (Fig. 2a). The division of the secondary cambium initiated taproot thickening in May (Fig. 2b, c) and divided continuously in July, which produced secondary phloem cells outward and secondary xylem cells inward that promoted rapid thickening of the taproot (Fig. 2d, e). From October, the thickening rate of the taproot slowed down and tended to stabilize (Fig. 2f, g). These results allowed us to identify four key stages associated with taproot thickening in *P. notoginseng*: seed root stage (March), initial thickening stage (May), rapid thickening stage (July) and stable thickening stage (November).

### RNA sequencing analysis of the developing taproot of *P. notoginseng*

**Fig. 3:**
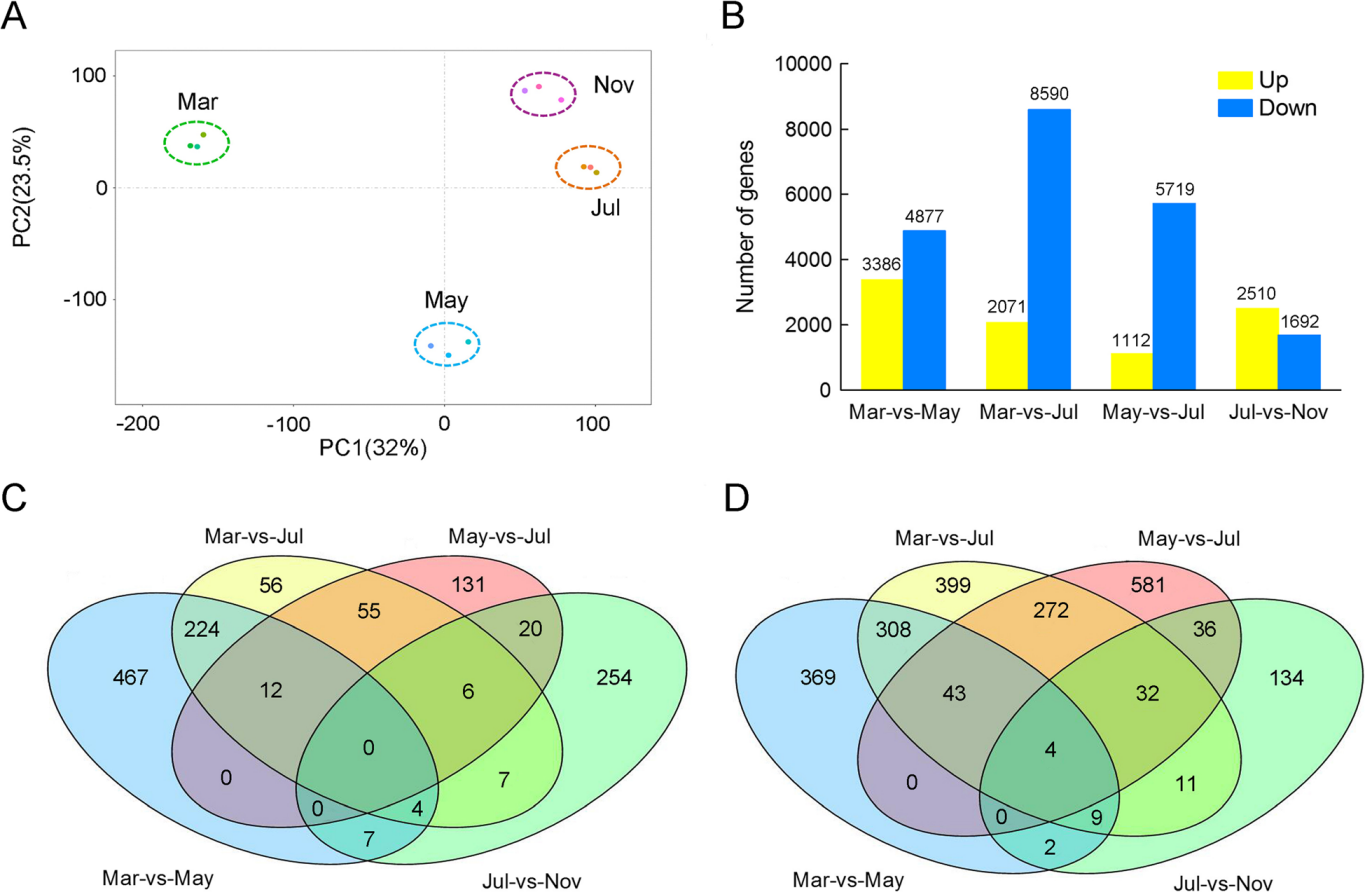
Global gene expression profiling of *P. notoginseng* taproots at different developmental stages. **a** Principal component analysis of the RNA-Seq data. **b** Numbers of detected differentially expressed genes (DEGs) among the four developmental stages (March, May, July, and November). **c**, **d** Venn diagrams of DEGs (FPKM≥10) among the four developmental stages

To explore the molecular basis of the morphological changes during taproot thickening, RNA sequencing (RNA-Seq) analysis of the taproots at four different stages was performed. Principal component analysis (PCA) revealed that the 12 samples could be clearly assigned to four groups. There was a significant difference between March and May, while July and November clustered together, suggesting that the overall transcriptomic profiles between the seed root and initial thickening stages were distinct, while those of the rapid and stable thickening stages were similar (Fig. 3a). In order to obtain reliable gene expression profiles, reads with log_2_ |Fold Change| > 1 and RPKM values > 10 were selected to annotate the differentially expressed genes (DEGs). A total of 9079 up-regulated DEGs and 20,878 down-regulated DEGs were derived from the comparison between the different stages. The number of down-regulated DEGs was higher than that of the up-regulated genes in March, May and July, whereas the opposite occurred in November (Fig. 3b). The up- or down-regulated DEGs in the different taproot thickening stages are listed in Additional file 1: Table S1. A Venn diagram of the DEGs showed that none were up-regulated consistently in May, July and November vs. March, while 12 genes were up-regulated consistently in May and July vs. March (Fig. 3c). By contrast, four genes were down-regulated consistently in May, July, and November vs. March, while 43 genes were down-regulated in May and July vs. March (Fig. 3d). As initial and rapid thickening are the key stages for taproot development, the consistently up-regulated or down-regulated genes in May and July vs. March play an important role in taproot development in *P. notoginseng*. In fact, the taproot almost stopped thickening in September (Fig. 1a). Therefore, the change in gene expression profile was not significant in the stable thickening stage, as reflected by the number of DEGs in November vs. July (Fig. 3b). The high-quality reads obtained in this study were deposited in the NCBI SRA database (accession number: SUB5611240).

**Fig. 4:**
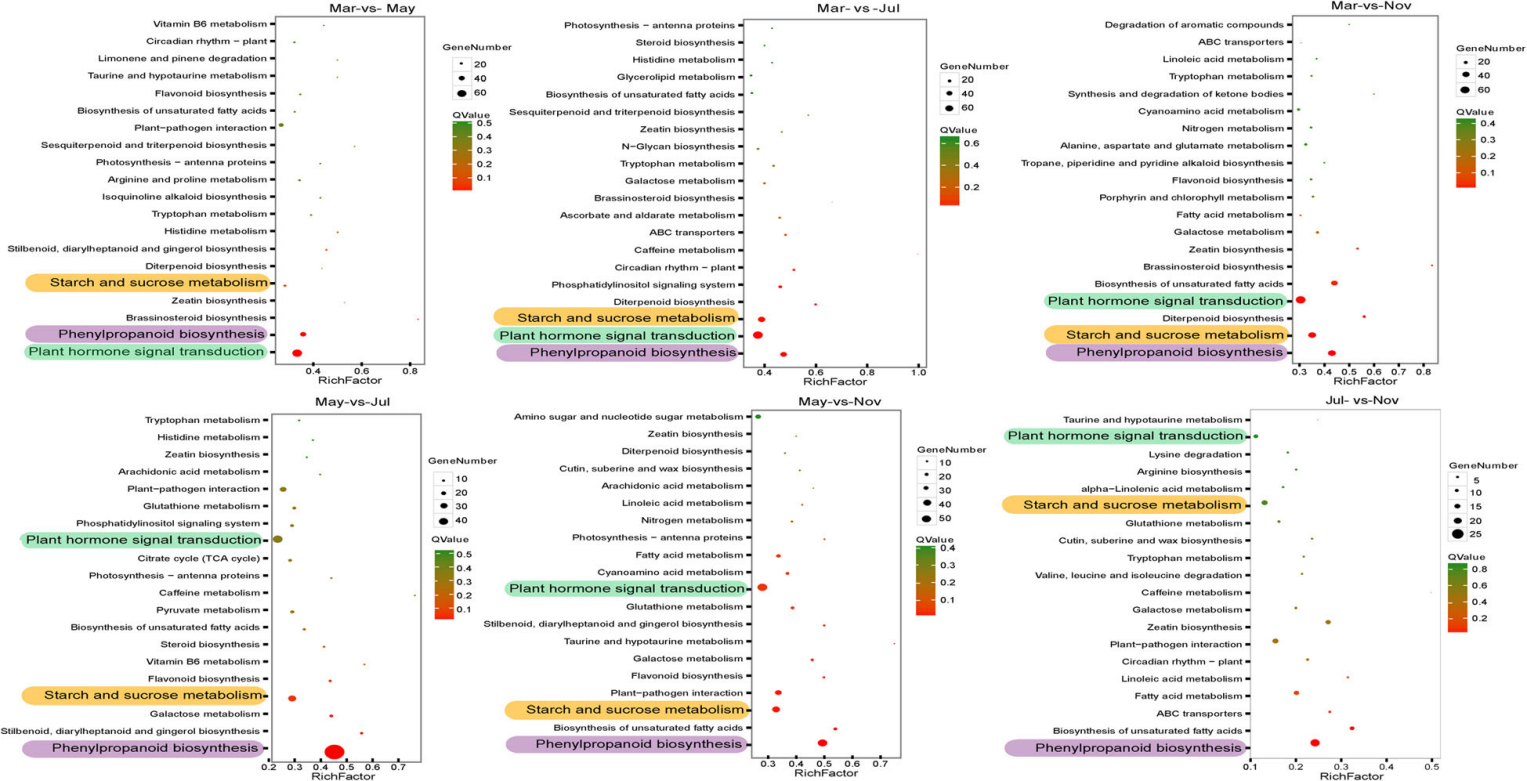
Significantly enriched KEGG pathways of DEGs. Top 20 significantly enriched KEGG pathways at different developmental stages. The Y-axis on the left represents KEGG pathways, and the X-axis indicates the “enrich factor” represented by the ratio of DEGs numbers to total annotated gene numbers of each pathway. Low q-values are shown in the red circle, and high q-values are shown in the green circle. The area of a circle represents DEGs number. The KEGG pathways, associated with the starch and sucrose metabolism, phenylpropanoid biosynthesis, and plant hormone signal transduction are underlined using the orange, purple, and aqua bar, respectively

The DEGs were further categorized and characterized according to the functional category defined by the Gene Ontology (GO) and Kyoto Encyclopedia of Genes and Genomes (KEGG) pathway. GO analysis showed that the DEGs were mainly involved in “metabolic process,” followed by “cell process” and “single-organism process” (Additional file 2: Figure S1). Metabolic processes analysis by KEGG showed that the DEGs were predominantly enriched in “plant hormone signal transduction pathway,” “starch and sucrose metabolism pathway,” and “phenylpropanoid biosynthesis pathway” (Fig. 4), suggesting that these pathways may be closely related to taproot thickening in *P. notoginseng*.

### Identifying critical genes controlling taproot development in *P. notoginseng*

According to the Venn diagram, 12 DEGs were significantly up-regulated in the pairwise comparisons among May vs. March, July vs. March, and July vs. May. These genes were mainly related to “carbohydrate and storage metabolism,” including genes encoding RNase-like major storage protein, Beta-amylase (BAMY) 5, Alpha-1,4 glucan phosphorylase L isozyme, Starch branching enzyme (SBE) I and Vacuolar protein sorting-associated protein 32 homolog 2, and “signal transduction and transcription,” including genes encoding Protein phosphatase 2C 37-like, Two-component response regulator-like APRR7, and DA1-related protein 5 (Table 1). In contrast, 43 down-regulated DEGs overlapped among the above-mentioned comparisons and were mainly involved in “signal transduction and transcription,” “stress/defense response,” and “other metabolisms,” including lipid metabolism, secondary metabolism, and cell wall metabolism (Table 2). Noticeably, four genes showing high homology with Delta(12)-fatty-acid desaturase FAD2-like (AtAT3G12120) were identified as consistently down-regulated during taproot thickening (Table 2). These results suggested that the genes associated with signal transduction, transcriptional regulation and metabolic pathways play diverse roles in taproot thickening in *P. notoginseng*.

**Table 1:** Identification of up-regulated DEGs at initial (May) and rapid (July) thickening stages of *P. notoginseng* taproots

ID	Description	TAIR ID	RPKM			
			Mar	May	Jul	Nov
Carbohydrate and storage metabolism						
Unigene0007855	RNase-like major storage protein	AT2G02990	22.3	3071.0	129,797.0	44,296.7
Unigene0036675	RNase-like major storage protein	AT2G02990	25.6	5736.2	140,415.0	48,725.4
Unigene0001769	Beta-amylase 5	AT4G15210	55.9	3345.0	11,618.7	4698.6
Unigene0035964	Alpha-1,4 glucan phosphorylase L isozyme	AT3G29320	8.4	302.2	728.3	570.6
Unigene0011567	Starch branching enzyme I	AT2G36390	18.4	259.8	1087.5	850.9
Unigene0036902	Vacuolar protein sorting-associated protein 32 homolog 2	AT4G29160	8.1	194.4	426.2	369.6
Signal transduction and transcription						
Unigene0032604	Protein phosphatase 2C 37-like	AT3G11410	11.9	19.1	53.7	77.2
Unigene0014980	Two-component response regulator-like APRR7	AT5G53420	20.5	39.0	209.9	90.9
Unigene0011239	DA1-related protein 5	AT5G62580	18.6	26.4	38.4	29.5
Secondary metabolism						
Unigene0034150	GBR5	/	208.3	357.5	810.6	601.6
Unigene0036783	Heterodimeric geranylgeranyl pyrophosphate synthase small subunit	AT4G38460	23.6	232.5	1157.4	282.1
Undefined						
Unigene0002182	Aspartate, glycine, lysine and serine-rich protein	AT5G20190	29.0	118.0	207.9	403.3

**Table 2:** Identification of down-regulated DEGs at initial (May) and rapid (July) thickening stages of *P. notoginseng* taproots

ID	Description	TAIR ID	RPKM			
			Mar	May	Jul	Nov
Signal transduction and transcription						
Unigene0046518	AP2/ERF domain-containing transcription factor DREB1D	AT5G51190	187.6	52.7	23.5	23.2
Unigene0019102	Ethylene-responsive transcription factor 2-like	AT4G17500	312.9	103.8	47.3	50.4
Unigene0036608	Auxin-responsive protein IAA7-like	AT3G23050	253.7	94.7	47.3	30.3
Unigene0039183	60 kDa jasmonate-induced protein-like	/	132.9	50.8	13.2	16.0
Unigene0018277	Calcium-binding protein PBP1-like	AT5G54490	107.9	50.6	19.4	19.6
Unigene0017010	Snakin-2-like	AT1G75750	152.6	60.2	16.9	9.0
Unigene0000195	Organ-specific protein S2-like	/	5774.5	2708.3	893.4	680.4
Unigene0024355	Organ-specific protein S2-like	/	2221.2	1084.2	307.9	264.8
Unigene0072131	ROTUNDIFOLIA like 7	AT3G55515	56.8	26.1	12.3	10.9
Unigene0053253	DUF793 domain-containing protein	AT1G01550	317.4	63.1	21.8	25.3
Unigene0020203	F-box protein	AT2G27310	283.0	99.5	43.6	42.7
Unigene0027395	Small ubiquitin-related modifier 2-A-like	AT5G55170	967.4	239.5	96.2	199.8
Unigene0035890	Lectin protein kinase family protein	AT5G24080	83.8	40.0	14.9	11.1
Stress/defense response						
Unigene0035676	Metallothionein-like protein 1	AT3G09390	991.4	287.2	97.9	192.2
Unigene0029269	Cysteine protease XCP2	AT1G20850	72.0	35.3	12.6	8.9
Unigene0027617	Pathogenesis-related protein 2	/	273.6	46.2	12.8	8.8
Unigene0020874	Metallothiol transferase FosB	/	62.9	26.3	13.0	13.5
Unigene0038257	Isocitrate dehydrogenase	AT1G65930	127.3	63.6	27.5	14.6
Lipid metabolism						
Unigene0039640	Delta(12)-fatty-acid desaturase FAD2-like	AT3G12120	100.5	44.8	20.2	7.6
*Unigene0010076	Delta(12)-fatty-acid desaturase FAD2-like	AT3G12120	188.0	68.9	28.5	12.5
*Unigene0053831	Delta(12) fatty acid desaturase FAD2-like	AT3G12120	686.0	327.6	113.8	53.0
*Unigene0014051	Delta(12)-fatty-acid desaturase FAD2-like	AT3G12120	438.8	215.2	77.5	25.0
Unigene0030153	Delta(12)-fatty-acid desaturase FAD2-like	AT3G12120	325.8	118.2	49.0	26.8
Unigene0070830	PI-PLC X-box domain-containing protein DDB_G0293730	AT4G38690	68.3	32.5	15.9	10.4
Unigene0036376	DUF563 domain-containing protein	AT2G41640	97.6	45.9	14.2	13.8
*Unigene0041208	CDP-diacylglycerol-glycerol-3-phosphate3-phosphatidyltransferase	AT3G48180	182.8	82.5	31.1	15.0
Unigene0022367	EXORDIUM-like 5	AT2G17230	156.7	70.4	14.6	28.3
Unigene0008870	GDSL-like Lipase/Acylhydrolase superfamily protein	AT3G26430	871.2	395.8	98.1	122.2
Secondary metabolism						
Unigene0034152	GBR5	/	2635.8	1186.3	411.3	465.4
Unigene0033007	Peroxidase 5-like	AT1G05260	219.3	40.2	15.8	29.5
Unigene0027454	Cytochrome P450 CYP72A219-like	AT3G14630	91.4	23.7	11.1	14.3
Unigene0045855	Caffeoyl-CoA O-methyltransferase	AT4G34050	219.5	76.9	37.6	27.5
Unigene0026392	Polyphenol oxidase	/	427.5	135.0	14.7	7.5
Unigene0040370	Shikimate O-hydroxycinnamoyltransferase-like	AT5G48930	87.1	38.5	12.1	8.4
Unigene0032140	Protein WALLS ARE THIN 1-like	AT1G75500	205.6	40.0	19.1	26.1
Cell wall metabolism						
Unigene0010296 Extensin-2-like		/	183.0	88.0	15.5	9.7
Unigene0038129 Extensin-3-like		AT3G09925	414.7	162.1	12.0	9.5
Transporter						
Unigene0054315	Copper-transporting ATPase RAN1-like (HMA7)	AT5G44790	58.1	25.9	12.2	26.3
Unigene0031786	High-affinity nitrate transporter 3.1-like	AT5G50200	290.4	58.0	21.4	29.0
Unigene0026653	Aquaporin TIP2–2	AT4G17340	578.2	166.1	26.4	56.6
Unigene0028381	Nodulin MtN21-like transporter family protein At5g07050-like	AT5G07050	55.2	26.5	11.2	12.1
Undefined						
Unigene0020809	Uncharacterized protein LOC108212493	AT2G47485	307.8	25.5	11.5	9.9
Unigene0025188	BnaC05g08810D [*Brassica napus*]	AT1G13360	128.0	44.2	13.0	23.4
Unigene0014035	Formin-like protein 18	AT1G10020	205.9	46.5	18.0	22.9
Unigene0047041	Plant organelle RNA recognition domain-containing protein	AT1G57680	136.9	67.3	32.8	95.6
Unigene0063477	Serine/arginine repetitive matrix protein 1	AT4G02040	59.9	25.4	11.6	14.1
Unigene0029630	Pyridoxal phosphate-dependent transferase	AT5G51920	74.9	29.1	13.1	10.7

* Asterisk indicates down-regulated DEGs among initial (May), rapid (July) and stable (November) thickening stages of *P. notoginseng* taproots

To validate the reliability of the transcriptomic data, we selected 16 DEGs that include 12 DEGs mentioned above for the quantitative real-time PCR (qRT-PCR) analysis. There was a good correlation (*r* = 0.83) between the transcriptomic data and the qRT-PCR results (Additional file 3: Figure S2). These results indicated that the transcriptomic data could reflect the transcriptional changes during the thickening process in the taproots of *P. notoginseng.*

### Differential expression of TFs

**Fig. 5:**
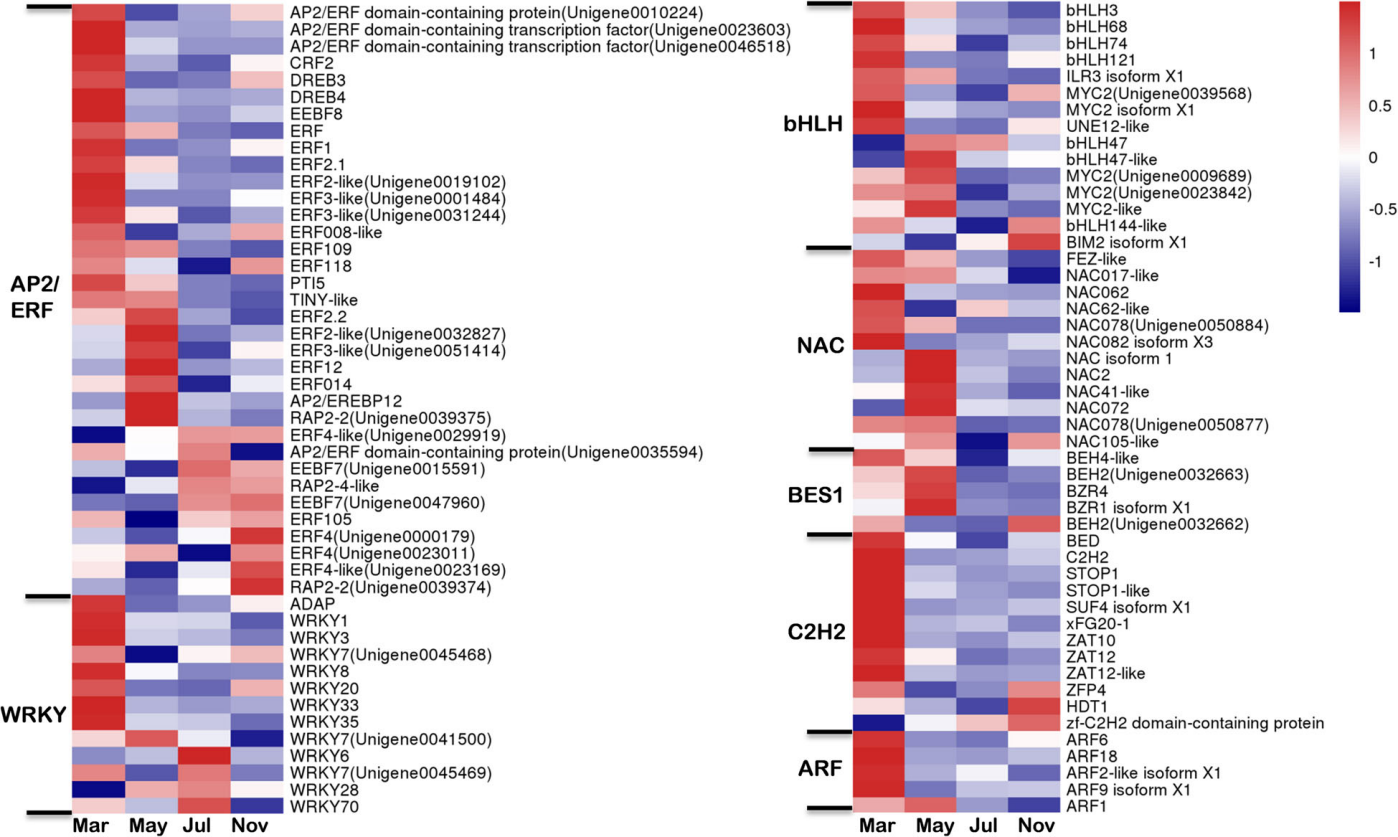
Expression patterns of DEGs assigned to transcription regulation in the four taproot transcriptomes, March, May, July, and November. The log-transformed expression values range from − 1 to 1. Red and blue colors indicate up- and down- regulated transcripts, respectively

A total of 237 DEGs were identified and assigned to 30 TF families (Additional file 4: Table S2). Among them, 97 DEGs encoding APETALA2/ethylene-responsive factor (AP2/ERF), WRKY, bHLH, NAC domain containing protein (NAC), BRI1-EMS-SUPPRESSOR 1 (BES1), Cys2-His2 zinc finger protein (C2H2), and Auxin response factor (ARF), have previously been implicated in plant growth and development [22–28] (Fig. 5). APETALA2/ethylene response factor (AP2/ERF) TF is one of the largest superfamilies in the plant kingdom, indicating that different members play a specific role in different taproot thickening stages (Fig. 5). Genes encoding the C2H2 and ARF families were predominantly up-regulated in March, while genes encoding the WRKY, bHLH, NAC, and BES1 families were up-regulated mainly in March and May (Fig. 5). These results suggest that the up-regulated TF families may be involved in the early developmental regulation of *P.notoginseng* taproot thickening.

### Differential expression of hormone signaling transcripts

**Fig. 6:**
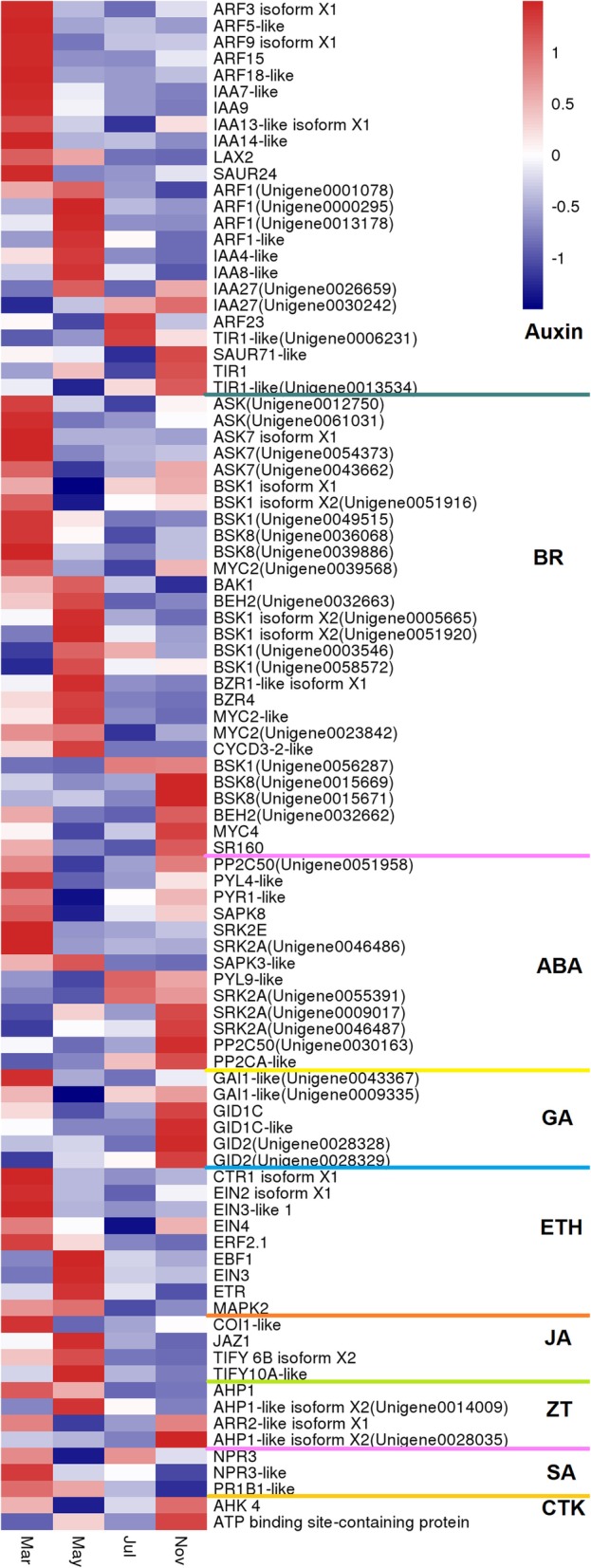
Expression patterns of DEGs assigned to hormone signal transduction in the four taproot transcriptomes, March, May, July, and November. The log-transformed expression values range from − 1 to 1. Red and blue colors indicate up- and down- regulated transcripts, respectively

The involvement of plant hormones, including indole-3-acetic acid (IAA), cytokinin (CTK), gibberellin (GA), ethylene (ETH), jasmonate (JA), and brassinosteroid (BR), in the development and formation of the roots has been investigated previously [29, 30]. Our KEGG analysis showed that “plant hormone signal transduction pathway” was predominantly enriched during taproot thickening in *P. notoginseng* (Fig. 4), with 93 DEGs grouped into the IAA, BR, abscisic acid (ABA), GA, ETH, JA, zeatin (ZT), salicylic acid (SA), and CTK pathways. While DEGs associated with the GA signaling pathway were up-regulated mainly in November, DEGs related to the ABA signaling pathway were significantly up-regulated in March and November (Fig. 6). To verify whether the gene expression was correlated with hormone metabolism, the endogenous IAA and JA contents at four taproot developmental stages were measured using liquid chromatography-mass spectrometry (LC-MS). The results showed that IAA and JA accumulated predominantly in March and May and both decreased significantly with the gradual thickening of the taproot, which is consistent with the transcriptome data (Additional file 5: Figure S3; Fig. 6). These results indicated that different hormones may have synergistic or/and antagonistic functions in the regulation of *P. notoginseng* taproot thickening.

### Differential expression of genes related to starch and sucrose metabolism

**Fig. 7:**
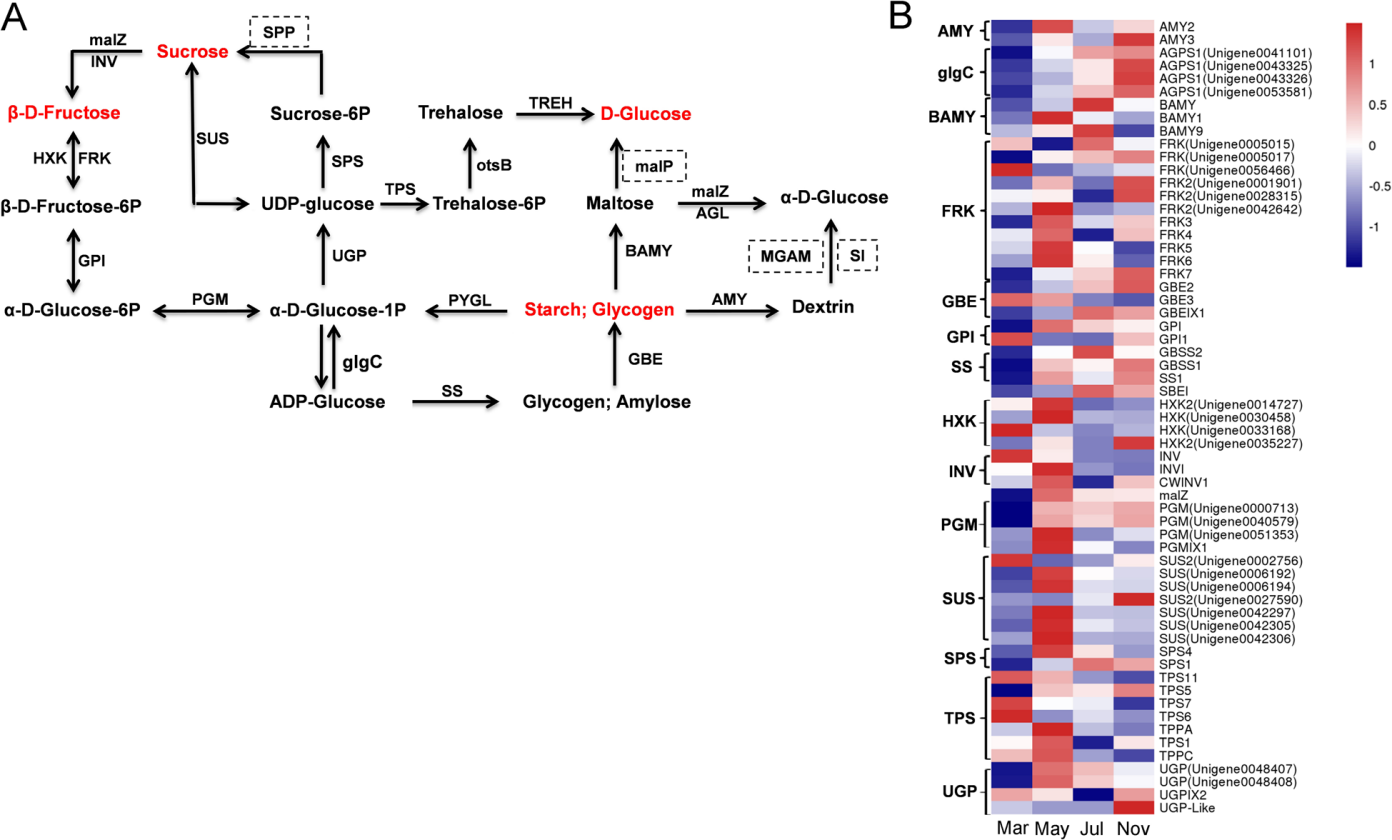
Starch and sucrose metabolic pathway (**a**) and expression patterns of DEGs assigned to starch and sugar metabolism in the four taproot transcriptomes, March, May, July, and November. **b**. The log-transformed expression values range from − 1 to 1. Red and blue colors indicate up- and down- regulated transcripts, respectively

**Fig. 8:**
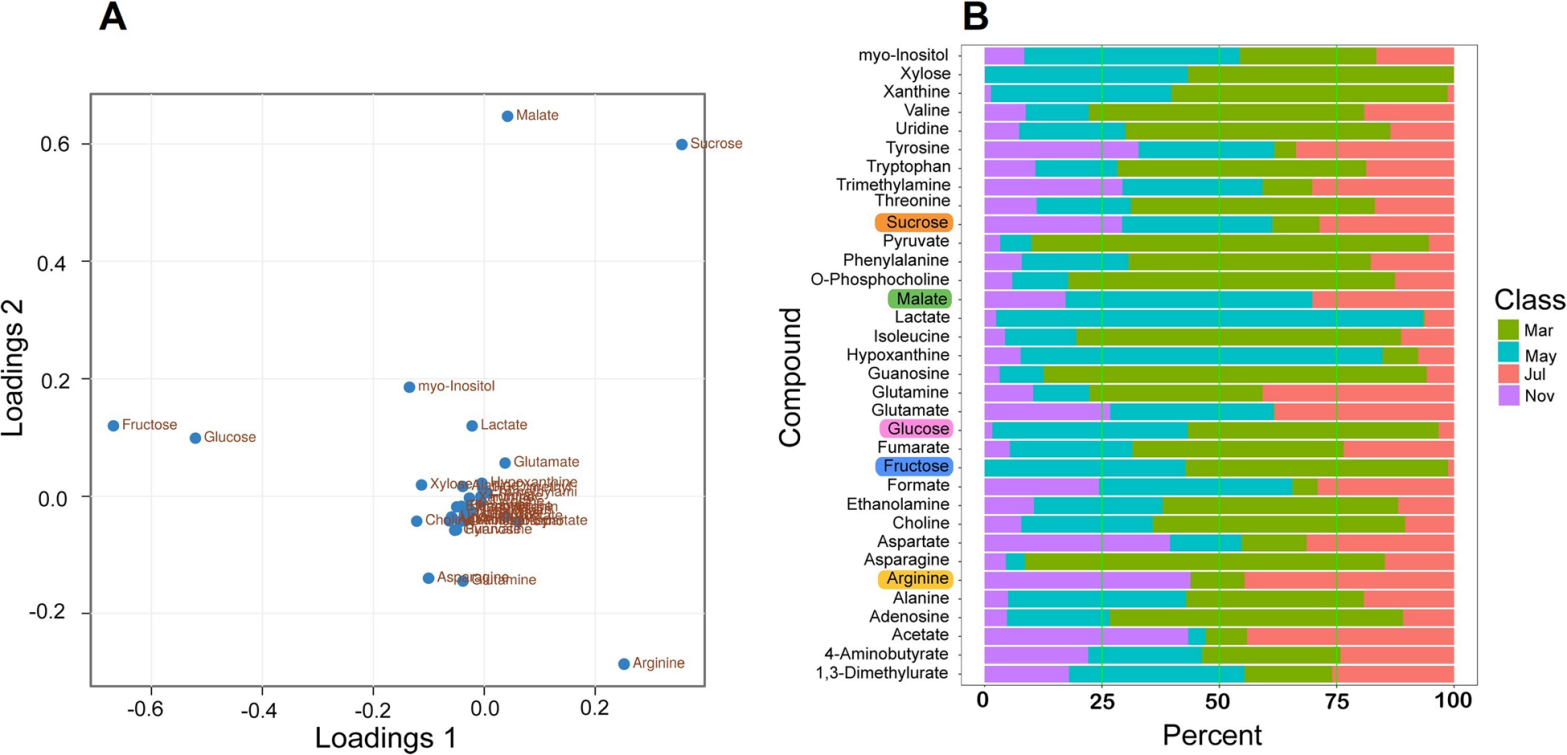
Expression profiles of primary metabolites during *P. notoginseng* taproot thickening. **a** Principal component analysis of the primary metabolites. **b** The change of primary metabolites in the four taproot stages, March, May, July, and November

The enrichment of DEGs related to “starch and sucrose metabolism” suggests their important role in *P. notoginseng* taproot thickening (Fig. 4). Metabolic pathway analysis showed that glucose, fructose, sucrose, and starch could be metabolically connected (Fig. 7a). Here, 61 DEGs encoding enzymes related to starch and sucrose metabolism were identified as up-regulated mostly in May, July, and November, including alpha-amylase genes (*AMY*), *BAMY*, starch synthase genes (*SS*), and granule-bound starch synthase genes (*GBSS*) involved in starch metabolism, and invertase genes (*INV*), sucrose synthase genes (*SuS*), and sucrose-phosphate synthase genes (*SPS*) involved in sucrose metabolism (Fig. 7b). To further clarify whether taproot thickening in *P. notoginseng* is directly associated with changes in carbohydrates, the primary metabolites at four taproot developmental stages were assessed by absolute quantitative analysis using Nuclear Magnetic Resonance (NMR) spectroscopy. PCA revealed that the four samples could be distinctly separated (Additional file 6: Figure S4). Samples collected from July and November clustered closely, suggesting that the overall metabolite profiles were similar between the rapid and stable thickening stages. The differential abundance profiles indicated that five metabolites, including fructose, glucose, sucrose, arginine and malate, differed significantly during taproot thickening (Fig. 8a). Among them, fructose and glucose accumulated dominantly in March and May, while sucrose accumulated mostly in May, July, and November. This was basically consistent with the transcriptome data (Fig.7b; Fig. 8a). It is presumed that sucrose is degraded into fructose and glucose for conversion into other organic matters during the early stage, while taproot thickening is also accompanied by increased malate and arginine accumulation (Fig. 8b).

### Differential expression of cell wall metabolism transcripts

**Fig. 9:**
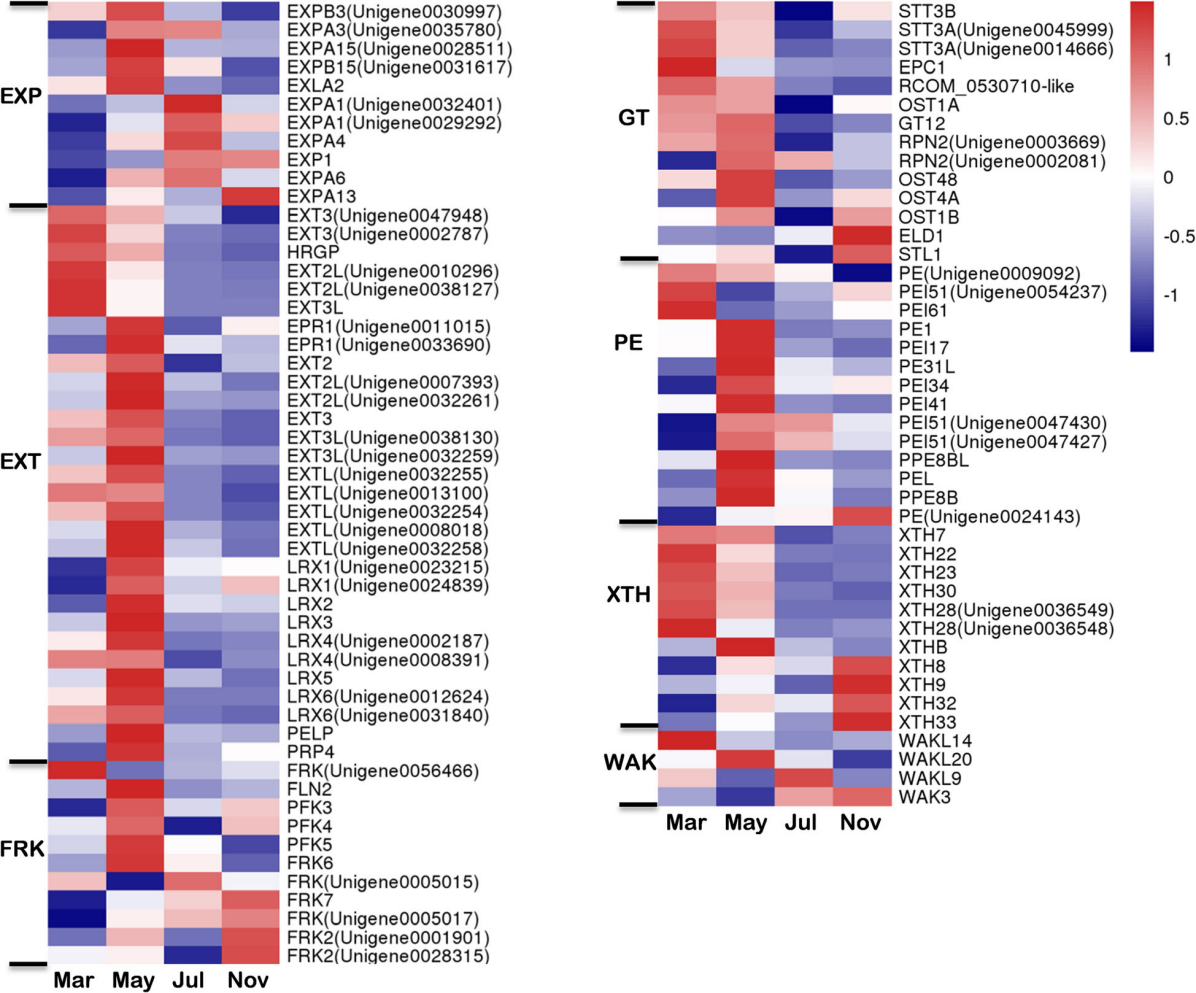
Expression patterns of DEGs assigned to cell wall metabolism in the four taproot transcriptomes, March, May, July, and November. The log-transformed expression values range from − 1 to 1. Red and blue colors indicate up- and down- regulated transcripts, respectively

The cell wall not only strengthens the plant body, but also has key roles in plant growth, cell differentiation, cell expansion, intercellular communication, water movement and defense [31, 32]. In the present study, a total of 96 DEGs encoding enzymes that are involved in cell wall modification, synthesis, and degradation were identified. Among them, the largest number of genes was up-regulated in May (Fig. 9), suggesting that the changes in cell wall components are necessary for the initiation and induction of taproot thickening in the early stages in *P. notoginseng*. In particular, DEGs encoding extension (EXT), glycosyltransferases (GT), and pectinesterase (PE) were mostly up-regulated in March and May, while those encoding expansin (EXP) were highly expressed in May and July (Fig. 9).

## Discussion

Elucidating the molecular mechanisms associated with taproot thickening is important for improving the yield and quality of *P. notoginsen*g products. Here, taproot development in one-year-old *P. notoginseng* plants at four critical stages was analyzed using an integrated morphological, transcriptomics, and metabonomics approach. We provided the framework for the gene expression changes involved in *P. notoginseng* taproot thickening, thus providing a foundation for the future elucidation of the molecular mechanisms underlying taproot growth and development in *P. notoginseng*.

### Potential DEGs playing critical roles in *P. notoginseng* taproot thickening

We found that taproot thickening in *P. notoginseng* was associated with vascular cambium activity, resulting in the production of the secondary xylem and phloem (Fig. 2). The further growth of the secondary xylem and secondary phloem is dependent on cell division, expansion, and differentiation and results in a rapid increase in root diameter; a process controlled by plant hormones, TFs, and metabolic pathways [33, 34].

### Hormonal signaling plays multidirectional roles in *P. notoginseng* taproot thickening

The plant hormone-mediated signaling pathway was the most significantly enriched pathway according to the KEGG pathway analysis (Fig. 4), as reflected by as many as 93 DEGs that were related to plant hormone signaling pathways (Fig. 6). Our study showed that the endogenous IAA content and genes involved in IAA signaling, including auxin/indole-3-acetic acid (Aux/IAAs) and auxin response factors (ARFs), were significantly up-regulated in the early stage and down-regulated in the later stage during *P. notoginseng* taproot thickening (Fig. 6; Additional file 5: Figure S3). These results suggested that IAA plays potential roles in the initial thickening of the *P. notoginseng* taproot, and degrading IAA and maintaining it at a lower concentration is necessary for promoting the continued thickening of the taproot. Congruent with our findings, previous studies in sweet potato (*Ipomoea batatas*) showed that auxin levels have been found to be elevated during early tuberous root formation [1, 35, 36]. The later stage of tuberous root development was positively correlated with concentrations of ABA and CK levels, but not with IAA levels [35].

It is unsurprising that the expression of genes related to the GA signaling pathway was significantly enhanced in November (Fig. 6). In general, GA is a negative regulatory hormone in storage organ formation and inhibits tuberization in potato [37]. Overexpression of the GA oxidase (GAox) gene could increase GA content, which promoted stolon elongation and inhibited storage organ formation [38]. Further research showed that *StPP2Ac2b*, a serine/threonine protein phosphatases type 2A (PP2A) from potato, acts in the stolons as a positive regulator of tuber induction by modulating the expression of *StGA2ox1* [39]. Xu et al. (1998) also observed that a high content of exogenous GA inhibited storage organ formation and promoted stolon elongation. Endogenous GA levels were high during stolon elongation and decreased when the stolon tips started to swell under inducing conditions in potato [40]. Our findings suggest that the initiation of thickening in the taproot of *P. notoginseng* is regulated by the crosstalk between IAA and GA.

In addition, the expression of genes related to the ABA signaling pathway was significantly enhanced in November (Fig. 6). In general, ABA accumulates during autumn. While it is possible that ABA-responsive genes are up-regulated in response to winter dormancy, it is known that ABA antagonizes GA, as demonstrated by the finding that ABA stimulates potato tuberization by counteracting GA [40–42]. In fact, recent studies have discovered the novel antagonizing roles of GA and ABA in integrating growth and development in plants with environmental signaling [43, 44]. These results suggest that antagonistic interactions between ABA and GA signaling potentially modulate taproot thickening in *P. notoginseng*.

### Transcriptional regulation is a basic regulatory factor for *P. notoginseng* taproot thickening

In *Arabidopsis*, ERF109, ERF114, and ERF115 were reported to positively regulate quiescent center (QC), distal stem cells (DSCs), and cell differentiation by mediating reactive oxygen species (ROS) signaling [45]. Here, a gene homologous to *Arabidopsis* ERF109 was significantly up-regulated in March and May (Fig. 5). Recent studies have further demonstrated that the highly JA-responsive ERF109 mediates the cross-talk between JA signaling and IAA biosynthesis to regulate root development in *Arabidopsis* [46, 47]. Moreover, JA induces the expression of ERF109 and ERF115, which regulates the asymmetric cell division of root stem cells and QC quiescence by the RETINOBLASTOMA-RELATED (RBR)-SCARECROW (SCR)-SHORT ROOT (SHR) protein network [47]. Interestingly, we also found that endogenous JA content and genes associated with JA signaling were significantly increased in the early stage of taproot development (Fig. 6; Additional file 5: Figure S3), suggesting that JA-induced ERF transcriptional regulation initiates the transition from seed roots to taproot thickening in *P. notoginseng*.

Furthermore, some genes encoding WRKY (e.g., WRKY3, 8), NAC (e.g., NAC1,2), MYC (e.g., MYC2), and ARF (e.g., ARF9) were up-regulated at the early stage of taproot development (Fig. 6). It was reported that MYC and NAC TFs might control secondary cell wall metabolism [48, 49]. Additionally, some NAC TFs have also been shown to be involved in the development of the secondary xylem [50, 51] and the regulation of the transition from cell division to cell expansion [52]. All these studies suggest that these TFs may be potentially involved in taproot thickening in *P. notoginseng*, but their exact roles require further exploration. In addition, we also found that a gene encoding DA1-related proteins was significantly up-regulated during the taproot thickening process (Table 1). DA1-related genes encode proteins containing a LIM domain and zinc finger-acting elements. In *Arabidopsis*, DAR2 functions in the regulation of root stem cell niche activity and root meristem size by maintaining the normal auxin distribution mediated by auxin transport, while DAR3 mediates proper root system architecture by unloading carbohydrates from the phloem to the root tip [53, 54], suggesting that the distribution of auxin rather than its content is more important for taproot thickening in *P. notoginseng.*

### Carbohydrate and storage protein metabolism provides materials for *P. notoginseng* taproot thickening

We found that “starch and sucrose metabolism pathways” was significantly activated during the development of the *P. notoginseng* taproot (Fig. 4). Sucrose cleavage is vital to multicellular plants for carbon metabolism and plant sink development. The reactions of SuS and INV catalyze sucrose cleavage, among which SuS can reversibly catalyze sucrose to UDP-glucose and fructose, while INV catalyzes sucrose to glucose (Glu) and fructose [55]. Here, genes encoding SuS and INV were mostly up-regulated in March and May (Fig. 7b), suggesting that sucrose catabolism is required for the thickening of *P. notoginseng* taproots. This was further confirmed by the discovery that glucose and fructose were abundantly accumulated, while sucrose was low, in March and May (Fig. 8b). Similar results were obtained in a previous study, and SuSy is a key enzyme involved in the early development of the storage root of radish [56].

Starch is considered to be one of the major storage carbohydrates. The accumulation of starch proceeds simultaneously with the swelling of the storage organs in potato, lotus (*Nelumbo nucifera*), and cassava [12, 37, 57]. Here, genes encoding SBE, GBSS, SS, and alpha-1,4-glucan phosphorylase L isozymes were significantly up-regulated in July and November (Fig. 7b; Table 1), implying that starch accumulation in an active sink plays a major role in the rapid and stable thickening stages. However, we also found that glucose and fructose were consumed in excess, while sucrose increased slightly, with taproot thickening (Fig. 8b), leading us to speculate that metabolic transition from starch to sucrose is required during the later stages of taproot thickening. This is based on the fact that genes encoding SPS were up-regulated in the rapid and stable thickening stages (Fig. 7b). This result is similar to that in previous studies on radish where up-regulated *SPS* was found to play a major role in the taproot thickening stage [8]. In fact, Jackson [58] noticed that a high content of sucrose is also a necessary condition during storage organ formation. In potato, sugars are thought to act as drivers of the formation and growth of the sink tuber [59]. These results indicate that the homeostasis and feedback regulation of starch and sucrose are required for the process of *P. notoginseng* taproot thickening, playing different roles at different thickening stages through dynamic metabolism.

Several lines of evidence suggest that storage proteins might be associated with the formation of underground storage organs [60–62]. Storage proteins that are localized in the vacuole and expressed mainly in the tuber or core have been reported in some species, such as patatin in potato and sporamin in sweetpotato [60]. In this study, genes encoding RNase-like major storage protein and vacuolar protein sorting-associated protein 32 homolog 2 were found to be significantly up-regulated during the process of *P. notoginseng* taproot thickening. (Table 1). A previous study showed that ginseng major protein (GMP) is a specific RNase-like storage protein in *Panax ginseng* roots and is thought to be highly correlated with the seasonally-regulated taproot development of *P. ginseng* [63]. Functional analysis of this candidate gene might be useful for the genetic engineering or marker-assisted selection of new *P. notoginseng* cultivars with enlarged taproots.

We also discovered that malate and arginine changed significantly during the *P. notoginseng* taproot thickening process (Fig. 8b). Malate, an important intermediate product of carbon metabolism in the citric acid cycle (TCA) and glyoxylic acid cycle, not only synthesizes some metabolites but also provides energy for plant metabolism [64]. Furthermore, it was also reported that malate can function as an intercellular diffusion factor, affecting the cell wall expansion of the roots [65]. Comparatively, arginine was mostly accumulated in the rapid and stable thickening stages (Fig. 8b). A recent study has shown that arginine produced by argininosuccinate lyase (ASL) is required for normal root elongation in rice, which is supported by the fact that the exogenous addition of arginine but not other amino acids rescued root growth in the *asl* mutant [66]. As the taproot of *P. notoginseng* was also significantly elongated during these two stages (Fig. 1b), we suspect that arginine might be involved in the taproot cell elongation of *P. notoginseng*. However, the specific acting mechanism is unclear, and there is only minor evidence that arginine is an essential substrate for the synthesis of protein and nitric oxide (NO, cell-signaling molecules) and the transport and storage of nitrogen in plants [67].

### The regulatory networks associated with *P. notoginseng* taproot thickening

**Fig. 10:**
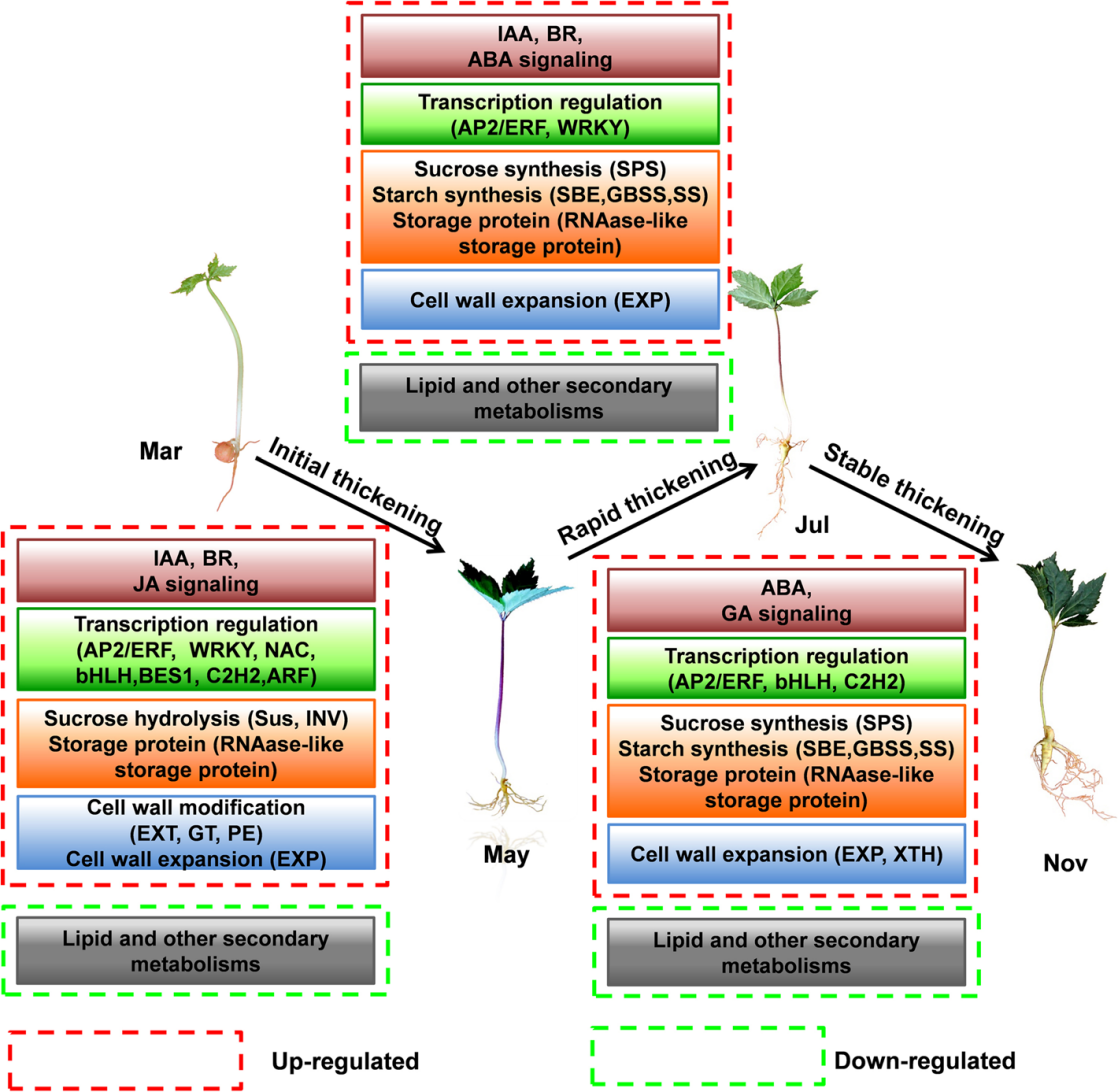
A proposed model of genetic and molecular interactions in the regulatory network during *P. notoginseng* taproot thickening

Taproot thickening in *P. notoginseng* is a complex regulatory process that is affected by many factors, including signal transduction pathways (hormone and transcription signaling) and metabolism processes (carbohydrate, storage, energy metabolism and cell wall). In this study, a putative model of the regulatory network associated with *P. notoginseng* taproot thickening was proposed by integrating both transcriptomics and metabonomics analysis with previous research findings. As illustrated in Fig. 10, hormones play a multidirectional role in the process of taproot thickening in *P. notoginseng*, involving the synergistic and antagonistic regulation of many hormones at different developmental stages. IAA mainly plays a role in the initial thickening stage. Persistent taproot thickening may be closely related to lower auxin levels and proper distribution, while GA may play a role in the later stage of taproot thickening by antagonizing ABA signaling. TFs mainly mediate transcriptional regulation in the initial thickening stage, and are closely associated with hormone signaling, such as early JA signaling mediation by ARFs. In the initial thickening stage, sucrose is hydrolyzed into fructose and glucose to synthesize starch for the rapid and stable thickening of the taproot, while the remaining glucose and fructose are re-synthesized into sucrose, which coordinates with starch to drive taproot thickening. Due to the prevailing sucrose-starch metabolism, lipids and other secondary metabolites (e.g., lignin) are down-regulated, thus coordinating the direct carbon flux into starch. In addition, storage proteins (e.g., RNase-like major storage protein), malate and arginine also play an important role in unknown functions during the taproot thickening process. Additionally, the modification and relaxation of the cell wall structure in the initial thickening stage contribute further to the continued thickening by *EXP*-mediated cell wall expansin. Taken together, these results implied that all these differentially expressed genes (DEGs) could play important roles in the regulatory network of *P. notoginseng* taproot thickening. Following further functional validation, these critical genes could greatly contribute to the manipulation of the shape, yield, and quality of the *P. notoginseng* taproot.

## Conclusions

Comparative transcriptome and metabolome changes at four critical developmental stages in the taproots of *P. notoginseng* were systematically investigated in this study. A total of 29,957 transcripts were obtained, and the DEGs during the taproot thickening process were identified for the first time. GO and KEGG pathway enrichment analysis revealed that these DEGs were mainly involved in processes of “plant hormone signal transduction,” “starch and sucrose metabolism,” and “phenylpropanoid biosynthesis.” Furthermore, integrated analysis of the DEGs, endogenous hormones, and primary metabolites provided a hypothetical model of the genetic regulatory network, highlighting that certain specific signal transduction pathways and metabolic processes are associated with taproot thickening in *P. notoginseng.* These findings not only potentially accelerate the process of genetic improvement of new *P. notoginseng* cultivars with enlarged taproots but also provide novel insights into the molecular regulatory mechanisms underlying root morphogenesis in this Chinese medicinal herb with enlarged roots.

## Methods

### Plant materials

One-year-old *P. notoginseng* plants at eight developmental stages, including March, May, June, July, August, September, October, and November were collected from the Xiaoxinzhai planting base (east longitude 104°24′29.0484“, north latitude 23°24’8.496”). They were planted by Wenshan Miao Xiang *P. notoginseng* Industrial Co., Ltd. For hormone detection, primary metabolite assays, and RNA-Seq, the collected seedlings were gently washed with clean water, and the shoots and fibrous roots were rapidly excised. The taproots were then placed in liquid nitrogen immediately and stored in an ultra-cold storage freezer at − 80 °C until further use.

### Determination of growth indexes and total saponin

The diameter of the taproot was measured using a Vernier caliper. The fresh weight of the taproots was weighed using an electronic balance, following which the dry weight was determined after drying for 3–5 days at 60 °C in an oven. For anatomical structure observation, taproots were cut into 2-mm-thick slices and immediately fixed in 5 mL formalin-glacial acetic acid-alcohol (FAA) solution for 48 h. Paraffin embedding was then performed as described by Canene-Adams [68]. For total saponin detection, dry taproots were ground into powder (through 80 mesh sieve), following which 0.6 g powder was added to 50 mL 70% methanol, left to stand for about 30 min, and then ultrasonicated for 30 min. The filtered samples were analyzed by high-performance liquid chromatography (HPLC). The chromatographic conditions were as follows: column (250 × 4.6, 5 m), acetonitrile (A) - water (B) as the mobile phase, detection wavelength 203 nm, and flow rate 1.5 mL/min.

### RNA library construction and sequencing

The taproots of one-year-old *P. notoginseng* plants collected in March, May, July, and November were used for RNA-Seq. Each sampling point had three biological replicates. After the total RNA was extracted, the eukaryotic mRNA was enriched by Oligo (dT) beads, while prokaryotic mRNA was enriched by removing rRNA by a Ribo-ZeroTM Magnetic Kit (Epicentre). The enriched mRNA was fragmented into short fragments using fragmentation buffer and then reverse transcribed into cDNA with random primers. Second-strand cDNA was synthesized by DNA polymerase I, RNase H, dNTPs, and buffer. The cDNA fragments were then purified using a QiaQuick PCR extraction kit, and following end-repair and the addition of poly(A) the fragments were ligated to the Illumina sequencing adapters. The ligation products were selected by agarose gel electrophoresis, PCR-amplified, and then sequenced using the Illumina HiSeqTM 4000 at Gene Denovo Biotechnology Co. (Guangzhou, China).

### Analysis of the RNA-Seq data

Clean data were obtained by removing reads containing adapters, poly-N, and low-quality reads from the raw data. The high-quality clean reads were mapped to ribosome RNA (rRNA) to identify residual rRNA reads. The high-quality clean reads were then mapped to the reference transcriptome using the short reads alignment tool Bowtie2 with the default parameters [69], and the mapping ratio was calculated. The gene abundances were calculated and normalized to RPKM (Reads Per kb per Million reads) [70]. To identify DEGs across samples or groups, the edgeR package (http://www.r-project.org/) was used. Reads with log2 |Fold Change| > 1, FDR < 0.05 and RPKM values > 10 were selected to annotate DETs. DEGs were then subjected to enrichment analysis of GO functions and KEGG pathways.

### Determination of endogenous IAA and JA contents

The taproots of one-year-old *P. notoginseng* plants collected in March, May, July and November were used to determine the IAA and JA contents. Standard calibration curves were constructed for IAA and JA to obtain two standard curve samples with concentrations ranging from 0.5 ng/mL to 1000 ng/mL. A mixed-standard solution containing 5 ng/mL of each of the deuterium-labeled plant hormones was prepared as the internal standard (IS) solution. A quality control (QC) sample was prepared by mixing plant hormone standards with the IS to obtain a final concentration of 10 ng/mL and 5 ng/mL. The QC sample was used to monitor the precision and stability of the instrument during the experiment runtime. One-hundred milligrams of sample was added to liquid nitrogen and 10 μL of IS was added to the sample in 1 mL extracting solution. The samples were vortexed for 30 s and placed in a shaker for 30 min in the dark. Two milliliters of dichloromethane was added to the mixture, which was then shaken in the dark for 30 min. Samples were centrifuged for 5 min at 4 °C at 3500 rpm. After carefully collecting the lower layer into a clean centrifuge tube, another 0.5 mL of dichloromethane was added to the sample and vortexed for 30 s. Samples were centrifuged for 5 min with the same settings as described above, and the lower layer was collected again. Both portions of the lower layer solution were combined, and solvent was evaporated under nitrogen. The purified samples were redissolved in 0.5 mL of 0.05% methanol and vortexed for 30 s. Solid particles were removed by first centrifuging at 3500 rpm for 10 min at 4 °C and then passing through a 0.22 μm filter. Filtrates were collected for LC-MS analysis.

### Primary metabolite analysis

The taproots of one-year-old *P. notoginseng* plants collected in March, May, July, and November were used to determine the primary metabolites by NMR. Briefly, freeze-dried plant material was weighed and suspended in a 1000 μL of 50%/50% methanol water solution. A 4 s on/off cycling program was used (eight cycles) for the in-solution ultrasonic extraction process (Sonics VX-130, USA). Samples were centrifuged at 13,000 rpm for 15 min, following which the supernatant was lyophilized and then re-dissolved in 450 μL of water. Four-hundred and fifty microliters of the aqueous layer was transferred to a clean 2 mL centrifuge tube following which 50 μL DSS standard solution (Anachro, Canada) was added. Samples were mixed well before being transferred to a 5 mm NMR tube (Norell, USA). Spectra were collected using a Bruker AV III 600 MHz spectrometer. The first increment of a 2D-1H, 1H-NOESY pulse sequence was utilized for the acquisition of ^1^H-NMR data and for suppressing the solvent signal. Experiments used a 100 ms mixing time along with a 990 ms pre-saturation (~ 80 Hz gammaB1). Spectra were collected at 25 °C, with a total of 128 scans over a period of 15 min.

### RT-qPCR analysis

Quantitative real-time PCR (RT-qPCR) was employed to validate the quality of RNA-seq results. Total RNAs from taproot samples at four stages including March, May, July, and November were extracted following the manufacturer’s instructions (Magen Biotech Co., Ltd., China) and reverse transcribed to cDNA following the manufacturer’s instructions (Vazyme Biotech Co.,Ltd., China). Each reaction was carried out using 10 μL 2× SYBR green reaction mix (Vazyme Biotech Co.,Ltd., China), 1.0 μL diluted cDNA, and 0.4 μL of each primer in a total volume of 20 μL system. RT-qPCR amplification reactions were performed in an ABI QuantStudio 5 Real-Time PCR System (Applied Biosystems). The specific primers used for RT-qPCR were designed with Primer3 web version 4.1.0 software, which are listed in Additional file 7: Table S3. Three technological replicates for each gene were performed and *P.notoginseng* *YLS8* was used as an endogenous control. The relative gene expression value was calculated with the 2^−ΔΔCT^ method.

## Supplementary information

**Additional file 1:** **Table S1.** The pairwise compared DEGs of four developmental stages in *P. notoginseng* taproots.

**Additional file 2:** **Figure S1.** Functional categories of up- and down-regulated unigenes at each developmental stage of *P. notoginseng* taproots. The classification was preformed according to gene ontology (GO) biological process.

**Additional file 3:** **Figure S2.** qRT-PCR verification diagram of DEGs during the thickening process in the taproots of *P. notoginseng*. **a** The expression levels determined by qRT-PCR and RNA-seq from four stages. **b** Correlation of gene expression ratio between RNA-Seq results (RPKM) and qPCR (2^-ΔΔCt)^ results. Results were calculated using log_2_ (Fold Change) values. The *r* value indicates the correlation coefficient. ** indicates significant difference at *p* ≤ 0.01.

**Additional file 4:** **Table S2.** Statistics analysis of TFs gene expression in taproot thickening in *P. notoginseng*.

**Additional file 5:** **Figure S3.** Change of endogenous indole-3-acetic acid (IAA) and jasmonate (JA) contents in taproot thickening in *P. notoginseng*. Results are shown as mean expression ± standard deviation of three independent experiments.

**Additional file 6:** **Figure S4.** Principal component analysis of primary metabolites in taproot thickening in *P. notoginseng*. Each sample contains five biological repeats.

**Additional file 7:** **Table S3.** Primers for qRT-PCR validation of candidate genes.

## Data Availability

All data generated or analyzed during this study are included in this published article and its supplementary information files.

## Abbreviations

ABA: Abscisic acid
ABC: ATP-bindingcassette transporter
AMY: Alpha-amylase genes
AP2/ERF: APETALA2/ethylene-responsive factor
APRR7: Arabidopsis PSEUDO-RESPONSE REGULATOR 7
ARF: Auxin response factor
ASL: Argininosuccinate lyase
Aux/IAA: Auxin/Indole-3-acetic acid
BAMY: Beta-amylase
BES: BRI1-EMS-suppressor
bHLH: Basic helixloop-helix transcription factor
BR: Brassinosteroid
bZIP: Basic leucine zipper
C2H2: Cys2-His2 zinc finger protein
CDC5: Cell division cycle 5-like protein
CLE: CLAVATA3/ESR-related gene
CTK: Cytokinin
DAR: DA1-related protein
DEG: Differentially expressed gene
DSC: Distal stem cells
ETH: Ethylene
EXP: Expansin
EXT: Extension
GA: Gibberellin
GAox: GA oxidase
GBE: 1,4-alpha-glucan-branching enzyme-like isoform X1
GBSS: Granule-bound starch synthase genes
GGR: Heterodimeric geranylgeranyl pyrophosphate synthase small subunit
GMP: Ginseng major protein
GO: Gene ontology
GT: Glycosyltransferases
HDS: 1-hydroxy-2-methyl-2-(E)-butenyl 4-diphosphate synthase
HMGR: 3-hydroxy-3-methylglutaryl-CoAreductase
IAA: Indole-3-acetic acid
INV: Invertase genes
JA: Jasmonate
JAZ1: Jasmonate ZIM-domain 1
KEGG: Kyoto encyclopedia of genesand genomes
LC-MS: Liquid chromatography-mass spectrometry
MVDD: Mevalonate diphosphate decarboxylase
MVK: Mevalonate kinase
NAC: NAM, ATAF, and CUC (NAC) transcription factors
NF-YA2: Nuclear transcription factor Y subunit A-2 protein
NMR: Nuclear Magnetic Resonance
NO: Nitric oxide
PE: Pectinesterase
PHO1: Alpha-1,4 glucan phosphorylase L isozyme
PMP: RNase-like major storage protein
PP2A: Serine/threonine protein phosphatases type 2A
QC: Quiescent center
RBR: Retinoblastoma-related
ROS: Reactive oxygen species
SA: Salicylic acid
SBE: Starch branching enzyme
SHR: Short root protein
SPS: Sucrose-phosphate synthase genes
SuS: Sucrose synthase genes
TCA: Tricarboxylic acid cycle
TF: Transcription factor
VPS32: Vacuolar protein sorting-associated protein 32 homolog 2
XTH: Xyloglucan endotransglucosylase/hydrolase protein
ZT: Zeatin
