# Supplementary material for: Comparative transcriptome and metabolome analyses provide new insights into the molecular mechanisms underlying taproot thickening in Panax notoginseng
Source: BMC Plant Biol. 2019 Oct 26;19:451. doi: 10.1186/s12870-019-2067-5 (PMC6815444; doi:10.1186/s12870-019-2067-5)
Supplement: Supplementary file 2 — Additional file 2: Figure S1. Functional categories of up- and down-regulated unigenes at each developmental stage of P. notoginseng taproots. The classification was preformed according to gene ontology (GO) biological process. [file 12870_2019_2067_MOESM2_ESM.docx]

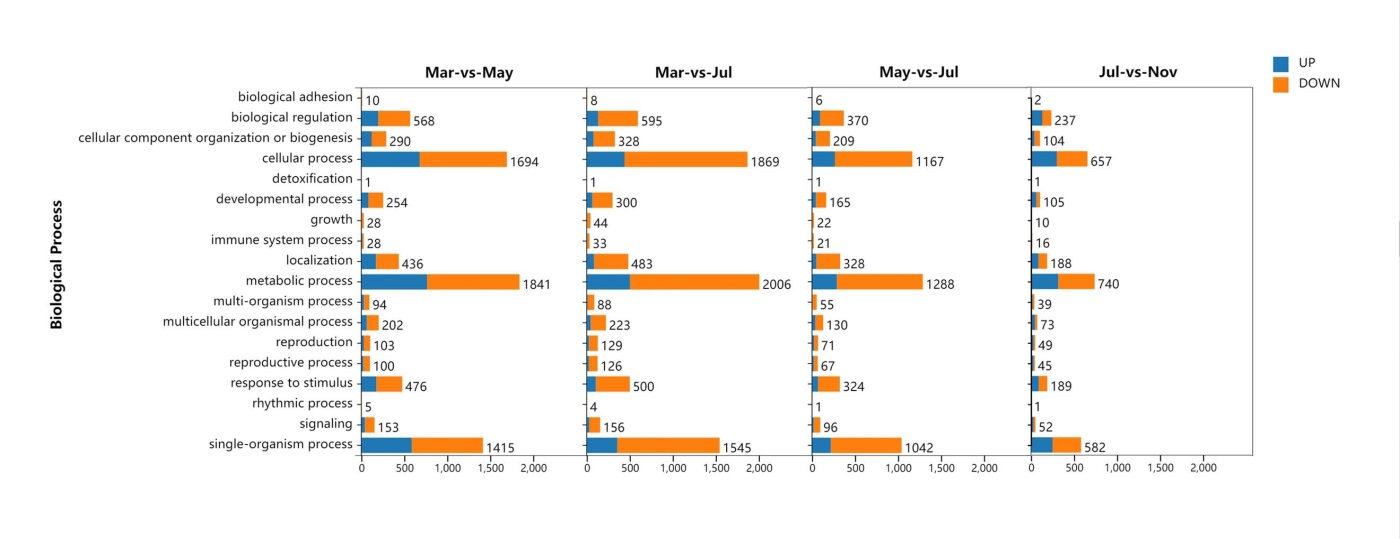


**Additional file 2：Figure S1.** Functional categories of up- and down-regulated unigenes at each developmental stage of *P.notoginseng* taproots. The classification was preformed according to gene ontology (GO) biological process.
