# Supplementary material for: Comparative transcriptome and metabolome analyses provide new insights into the molecular mechanisms underlying taproot thickening in Panax notoginseng
Source: BMC Plant Biol. 2019 Oct 26;19:451. doi: 10.1186/s12870-019-2067-5 (PMC6815444; doi:10.1186/s12870-019-2067-5)
Supplement: Supplementary file 3 — Additional file 3: Figure S2. qRT-PCR verification diagram of DEGs during the thickening process in the taproots of P. notoginseng. a The expression levels determined by qRT-PCR and RNA-seq from four stages. b Correlation of gene expression ratio between RNA-Seq results (RPKM) and qPCR (2-ΔΔCt) results. Results were calculated using log2 (Fold Change) values. The r value indicates the correlation coefficient. ** indicates significant difference at p ≤ 0.01. [file 12870_2019_2067_MOESM3_ESM.docx]

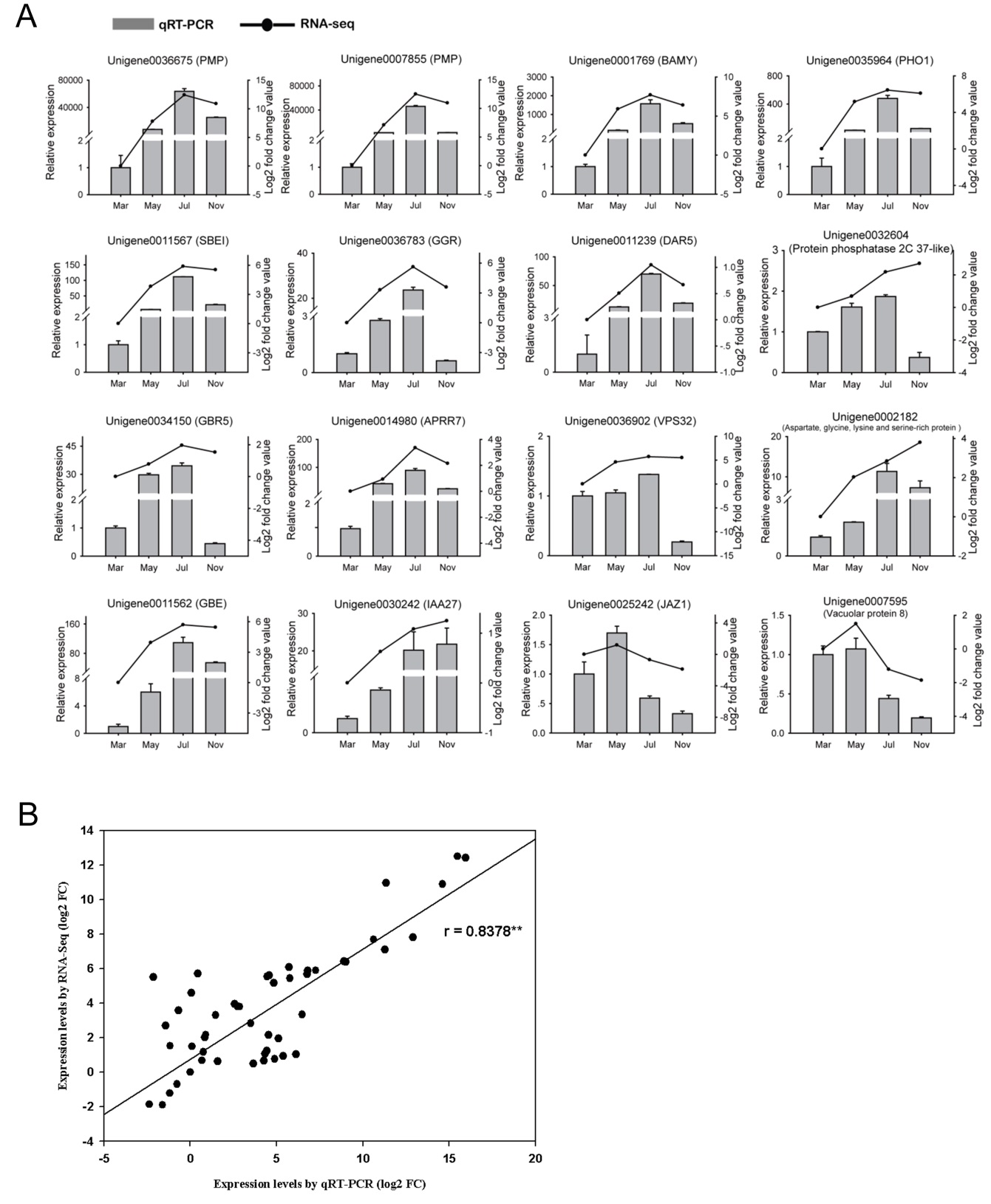


**Additional file 3: Figure S2.** qRT-PCR verification diagram of DEGs during the thickening process in the taproots of P. notoginseng. **a** The expression levels determined by qRT-PCR and RNA-seq from four stages. **b** Correlation of gene expression ratio between RNA-Seq results (RPKM) and qPCR (2^-ΔΔCt)^ results. Results were calculated using log_2_ (Fold Change) values. The *r* value indicates the correlation coefficient. ** indicates significant difference at p ≤ 0.01.
