# Supplementary material for: Comparative transcriptome and metabolome analyses provide new insights into the molecular mechanisms underlying taproot thickening in Panax notoginseng
Source: BMC Plant Biol. 2019 Oct 26;19:451. doi: 10.1186/s12870-019-2067-5 (PMC6815444; doi:10.1186/s12870-019-2067-5)
Supplement: Supplementary file 4 — Additional file 4: Table S2. Statistics analysis of TFs gene expression in taproot thickening in P. notoginseng. [file 12870_2019_2067_MOESM4_ESM.docx]

**Additional file 4: Table S2.** Statistics analysis of TFs gene expression in taproot thickening in *P. notoginseng*

| **Transcription factor family** | **Total** | **Mar-vs-May** | | **Mar-vs-Jul** | | **May-vs-Jul** | | **Jul-vs-Nov** | |
| --- | --- | --- | --- | --- | --- | --- | --- | --- | --- |
|  |  | **up** | **down** | **up** | **down** | **up** | **down** | **up** | **down** |
| ERF | 37 | 11 | 26 | 9 | 28 | 14 | 23 | 25 | 12 |
| C3H | 18 | 3 | 15 | 3 | 15 | 9 | 9 | 13 | 5 |
| bHLH | 17 | 6 | 11 | 4 | 13 | 1 | 16 | 11 | 6 |
| NAC | 14 | 6 | 8 | 5 | 9 | 4 | 10 | 5 | 9 |
| C2H2 | 12 | 1 | 11 | 1 | 11 | 5 | 7 | 10 | 2 |
| WRKY | 12 | 3 | 9 | 4 | 8 | 5 | 7 | 4 | 8 |
| bZIP | 12 | 4 | 8 | 2 | 10 | 2 | 10 | 7 | 5 |
| Trihelix | 11 | 5 | 6 | 4 | 7 | 8 | 3 | 10 | 1 |
| HD-ZIP | 11 | 6 | 5 | 4 | 7 | 7 | 4 | 9 | 2 |
| GRAS | 10 | 2 | 8 | 1 | 9 | 4 | 6 | 9 | 1 |
| MYB | 8 | 2 | 6 | 5 | 3 | 6 | 2 | 7 | 1 |
| MYB_related | 7 | 5 | 2 | 5 | 2 | 4 | 3 | 6 | 1 |
| Dof | 7 | 4 | 3 | 4 | 3 | 2 | 5 | 5 | 2 |
| GATA | 6 | 0 | 6 | 2 | 4 | 3 | 3 | 5 | 1 |
| HSF | 6 | 4 | 2 | 3 | 3 | 3 | 3 | 1 | 5 |
| ARF | 6 | 1 | 5 | 0 | 6 | 3 | 3 | 4 | 2 |
| G2-like | 5 | 1 | 4 | 1 | 4 | 2 | 3 | 3 | 2 |
| SBP | 5 | 0 | 5 | 0 | 5 | 3 | 2 | 5 | 0 |
| BBR-BPC | 5 | 0 | 5 | 1 | 4 | 2 | 3 | 5 | 0 |
| BES1 | 5 | 3 | 2 | 0 | 5 | 0 | 5 | 3 | 2 |
| TALE | 4 | 2 | 2 | 2 | 2 | 4 | 0 | 2 | 2 |
| TCP | 3 | 2 | 1 | 2 | 1 | 3 | 0 | 2 | 1 |
| NF-YB | 3 | 2 | 1 | 1 | 2 | 2 | 1 | 3 | 0 |
| NF-YC | 3 | 1 | 2 | 2 | 1 | 2 | 1 | 1 | 2 |
| LBD | 2 | 0 | 2 | 0 | 2 | 2 | 0 | 1 | 1 |
| ARR-B | 2 | 0 | 2 | 0 | 2 | 2 | 0 | 2 | 0 |
| WOX | 2 | 1 | 1 | 1 | 1 | 2 | 0 | 1 | 1 |
| CO-like | 2 | 1 | 1 | 1 | 1 | 0 | 2 | 2 | 0 |
| E2F/DP | 1 | 0 | 1 | 0 | 1 | 0 | 1 | 1 | 0 |
| AP2 | 1 | 0 | 1 | 0 | 1 | 1 | 0 | 1 | 0 |
