# Supplementary material for: Comparative transcriptome and metabolome analyses provide new insights into the molecular mechanisms underlying taproot thickening in Panax notoginseng
Source: BMC Plant Biol. 2019 Oct 26;19:451. doi: 10.1186/s12870-019-2067-5 (PMC6815444; doi:10.1186/s12870-019-2067-5)
Supplement: Supplementary file 5 — Additional file 5: Figure S3. Change of endogenous indole-3-acetic acid (IAA) and jasmonate (JA) contents in taproot thickening in P. notoginseng. Results are shown as mean expression ± standard deviation of three independent experiments. [file 12870_2019_2067_MOESM5_ESM.docx]

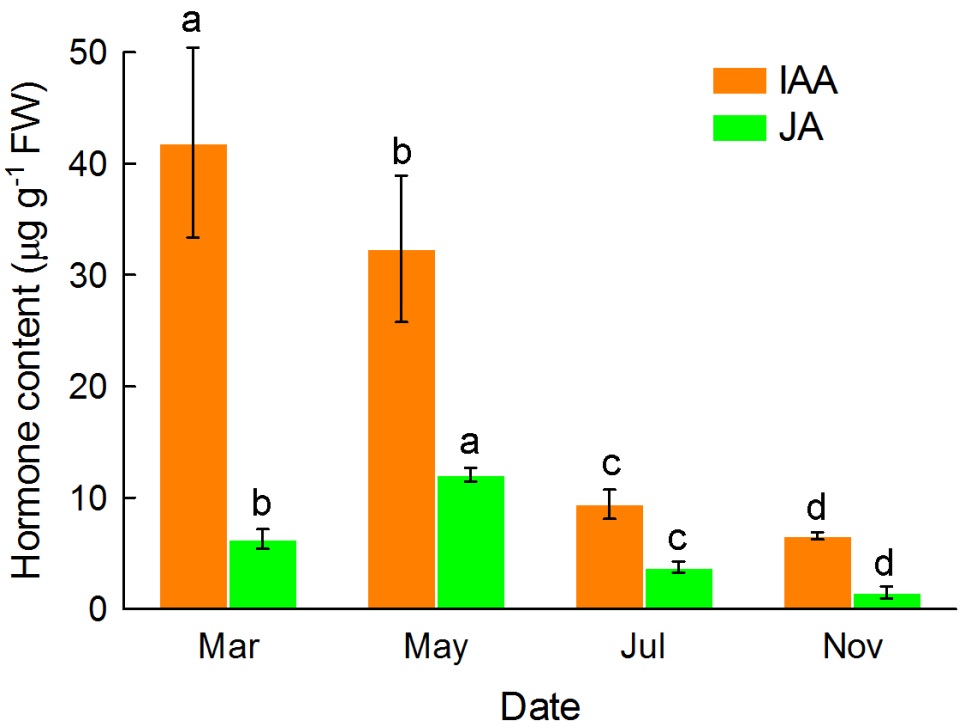


**Additional file 5: Figure S3.** Change of endogenous indole-3-acetic acid (IAA) and jasmonate (JA) contents in taproot thickening in *P. notoginseng*. Results are shown as mean expression ± standard deviation of three independent experiments.
