# Supplementary material for: Comparative transcriptome and metabolome analyses provide new insights into the molecular mechanisms underlying taproot thickening in Panax notoginseng
Source: BMC Plant Biol. 2019 Oct 26;19:451. doi: 10.1186/s12870-019-2067-5 (PMC6815444; doi:10.1186/s12870-019-2067-5)
Supplement: Supplementary file 6 — Additional file 6: Figure S4. Principal component analysis of primary metabolites in taproot thickening in P. notoginseng. Each sample contains five biological repeats. [file 12870_2019_2067_MOESM6_ESM.docx]

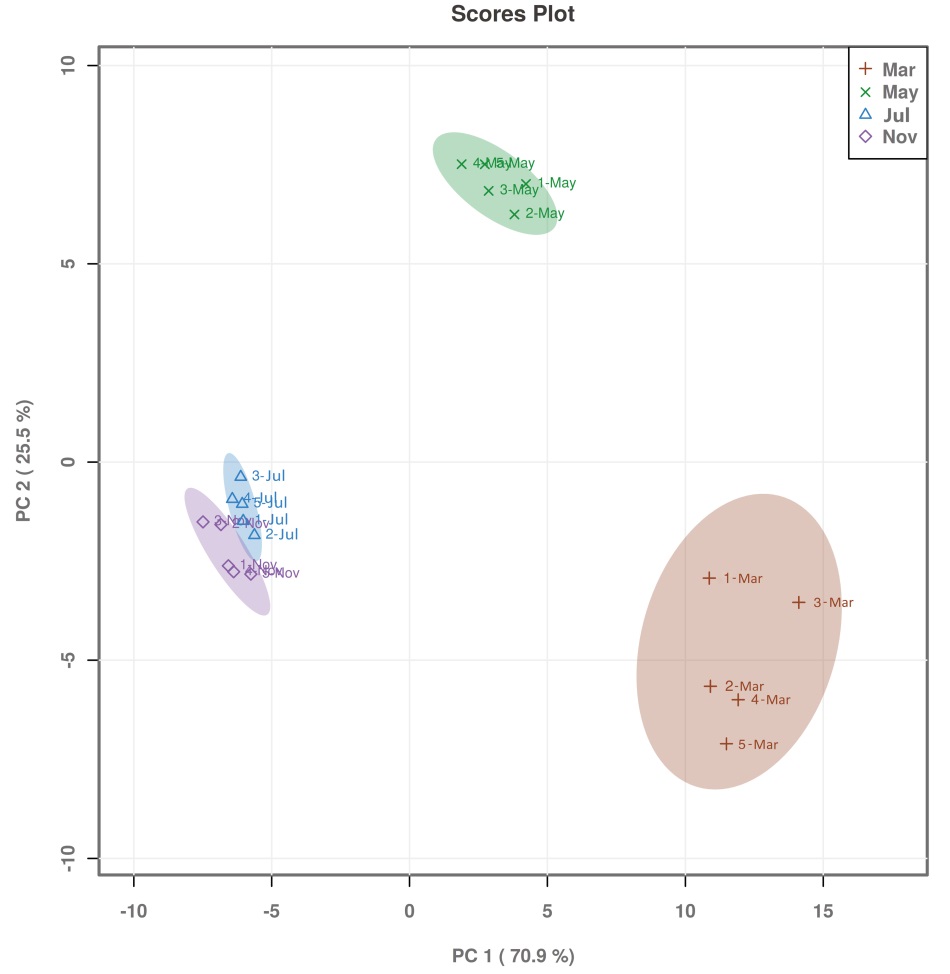


**Additional file 6: Figure S4.** Principal component analysis of primary metabolites in taproot thickening in *P. notoginseng*. Each sample contains five biological repeats.
