# Supplementary material for: Comparative transcriptome and metabolome analyses provide new insights into the molecular mechanisms underlying taproot thickening in Panax notoginseng
Source: BMC Plant Biol. 2019 Oct 26;19:451. doi: 10.1186/s12870-019-2067-5 (PMC6815444; doi:10.1186/s12870-019-2067-5)
Supplement: Supplementary file 7 — Additional file 7: Table S3. Primers for qRT-PCR validation of candidate genes. [file 12870_2019_2067_MOESM7_ESM.docx]

**Additional file 7：Table S3.** Primers for qRT-PCR validation of candidate genes.

|  | **Primer** | **Primer sequence** | **Primer** | **Primer sequence** |
| --- | --- | --- | --- | --- |
| 1 | YLS8-F | GATTGATCTTGGCACCGGGA | YLS8-R | TGACCAACCCACGACCTTTC |
| 2 | Unigene0036675-F | AGAACGGCGCTTGCTTTTAG | Unigene0036675-R | AGTAGCGTCATCTGTAGGGC |
| 3 | Unigene0007855-F | AATTCTGGGAACACGAGTGG | Unigene0007855-R | TTTCTGGCACAGCATTCAAG |
| 4 | Unigene0001769-F | GTGAACAGACGCCCACAAAT | Unigene0001769-R | TGGGAATGGCTTGATGGGAT |
| 5 | Unigene0035964-F | AGACACTGGACTGGTGGAGA | Unigene0035964-R | CTTCACAGGCTTTGGTGTGC |
| 6 | Unigene0011567-F | GGGCCATGATGCGGACTATT | Unigene0011567-R | CATGTTCGAGCCGGTGAGAG |
| 7 | Unigene0036783-F | TCACCGGACGATGCCATATC | Unigene0036783-R | GTACGCGTGGTGAATTGTGG |
| 8 | Unigene0011239-F | TCATGGCCTAGAGCTGACCT | Unigene0011239-R | TTGCACCAGATGCAGAGAAC |
| 9 | Unigene0032604-F | CGATGATGCGGAGCTTTTCG | Unigene0032604-R | TGATCGGTGGACAGAGGAGT |
| 10 | Unigene0034150-F | CAAAAATGACGGACATCACG | Unigene0034150-R | GGTAGTCTCTGCCTCCACCA |
| 11 | Unigene0014980-F | TTGCAACGGATGAAAGCCCT | Unigene0014980-R | TCCCATAAACAGCCCCGAAG |
| 12 | Unigene0036902-F | GACGAGTTGGAGGCAGAACT | Unigene0036902-R | TTGCTTGCTAGCGTGGACAG |
| 13 | Unigene0002182-F | CACAAGGATTCCGCTCGTG | Unigene0002182-R | CAGGGAAGAAACTGGGTTGC |
| 14 | Unigene0011562-F | AGTTGTGGGATAGCAGGCTT | Unigene0011562-R | TATAGTCCCCTGTGAACGCC |
| 15 | Unigene0030242-F | TTCTCTGGTAGTGGCGGATC | Unigene0030242-R | CCCATGGCCATTTGTAGCTG |
| 16 | Unigene0025242-F | GCGGTCAAGTCATCGTGTTC | Unigene0025242-R | CGCGCTCTTGGATTATGCTG |
| 17 | Unigene0007595-F | CAGCTTCCAATTCAACCGCT | Unigene0007595-R | CCCTTTCTTGCAATCCCACC |
